# Opportunities and Challenges of Extracting Values in Autobiographical Narratives

**DOI:** 10.3389/fpsyg.2022.886455

**Published:** 2022-08-02

**Authors:** Ronald Fischer, Johannes Karl, Velichko Fetvadjiev, Adam Grener, Markus Luczak-Roesch

**Affiliations:** ^1^School of Psychology, Victoria University of Wellington, Wellington, New Zealand; ^2^Unidade de Neurociências Cognitiva e Neuroinformática (Cognitive Neuroscience and Neuroinformatics Unit), Instituto D'Or de Pesquisa e Ensino, Rio de Janeiro, Brazil; ^3^School of Psychology, Dublin City University, Dublin, Ireland; ^4^Faculty of Social and Behavioural Sciences, University of Amsterdam, Amsterdam, Netherlands; ^5^North-West University, Potchefstroom, South Africa; ^6^School of English, Film, Theatre, Media Studies, and Art History, Victoria University of Wellington, Wellington, New Zealand; ^7^School of Information Management, Victoria University of Wellington, Wellington, New Zealand

**Keywords:** lexical analysis, values, autobiographical stories, natural language processing, life story method, text mining, implicit motives

## Abstract

We report three studies in which we applied a value dictionary to narratives. Our objective was to test a theory-driven value dictionary for extracting valuable information from autobiographical and narrative texts. In Studies 1 (*N* = 106) and 2 (*N* = 152), participants wrote short autobiographical narratives and in Study 3 (*N* = 150), participants wrote narratives based on ambiguous stimuli. Participants in all three studies also completed the Portrait Value Questionnaire as a self-report measure of values. Overall, our results demonstrate that it is possible to extract value-relevant information from these narratives. Extracted values from autobiographical narratives showed average correlations of 0.07 (Study 1) and 0.12 (Study 2) with self-reports compared to an average correlation of 0.01 for the extracted values from implicit motive tasks (Study 3). The correlations with self-reports were in line with previous validation studies. The most salient values in narratives diverged somewhat, with a stronger emphasis on achievement values compared to self-reports, probably due to the nature of salient episodes within one's life that require demonstrating success according to social standards. Benevolence values were consistently most important in both self-ratings and text-based scoring. The value structure emerging from narratives diverged from the theoretically predicted structure, yet broad personally vs. socially focused value dimensions were qualitatively discernible. We highlight opportunities and challenges for future value research using autobiographical stories.

## Introduction

The study of values is fundamental for understanding the goals and motivations of individuals because values refer to trans-situationally important goals in a person's life that guide the thoughts and behavior and provide standards for evaluating actors, events, and situations (Schwartz and Bilsky, 1987). Some of the earliest attempts to identify human values have been based on linguistic data (Allport and Odbert, 1936), but the study of values have been dominated by self-report measures, most notably those based on Schwartz's seminal theory of values (Schwartz, 1992; Schwartz et al., 2001, 2012). Using these surveys, values have been shown to relate to personality traits, attitudes, moral issues, and behavior across a range of contexts (Boer and Fischer, 2013; Fischer and Boer, 2015; Fischer, 2017; Roccas and Sagiv, 2017). The reliance of value research on self-reports certainly limits the opportunities for analyzing values at a distance, across time and space, which is increasingly becoming possible through data that are being produced in social media contexts (Ponizovskiy et al., 2020; Boyd et al., 2021). There have been manual coding approaches in which human coders analyzed texts at a deep level to infer salient values expressed by the authors (Suedfeld et al., 2010, 2011; Portman, 2014). Ponizovskiy et al. (2020) recently developed a theory-based value dictionary that overcomes two major limitations of such previous indirect measures of values, that is, the lack of validated dictionaries and the effort that is involved in manual coding of texts. This theory-based dictionary has been developed and validated in the context of social media texts, using various types of blogs, Facebook updates and value-focused texts are produced by MTurk workers and therefore offers a promising tool for further value research.

We expand this study in a number of ways. First, we tested to find whether values can be extracted from short autobiographic stories with this recently developed theory-based dictionary. Autobiographical research has suggested that individuals build coherent narratives around their values and beliefs (McAdams et al., 2004; McAdams and Pals, 2006). Therefore, it is worth exploring to what extent salient values of individuals can be extracted from autobiographical stories.

Second, an older tradition of research attempted to extract person-relevant information from individuals' responses to ambiguous stimuli as an indication of implicitly held motives (Schultheiss and Pang, 2007). Previous studies suggested that implicit motive measures do not converge strongly with explicit value ratings (Hofer et al., 2006). We have been examining whether values extracted from stories elicited using implicit assessment techniques correlate with self-reports to a comparable degree as values extracted from autobiographical stories. Using both autobiographical stories and implicit stories based on ambiguous stimuli material, we can provide some benchmarks on possible text-based value analyses with a theory-based dictionary.

A final contribution is a closer examination of the values and their associations as identified through textual analysis. To what extent are values organized in the same circular space based on motivational compatibilities and conflicts as has been described by Schwartz? Recent studies examining values as situational states (instead of more stable trait-like structures as is common in value research) have suggested that values may be organized differently when considered in the context of specific situations compared to values as relevant to life in general (Skimina et al., 2021). Narratives or stories are often about the descriptions of specific events; hence, they may be more like states than traits. At the same time, the request to reflect on salient episodes within one's life may motivate individuals to provide more abstract reflections on specific events which may be compatible with value traits. Hence, it is theoretically interesting to explore the structure of values in the context of personally meaningful life events.

### Schwartz's Nearly Universal Theory of Values

Schwartz (1992) presented a descriptive theory of human values based on their motivational compatibilities and conflicts. Individual values are thought to be motivational goals that show mutual conflicts and congruence which result in a two-dimensional structure when considering the overall levels of compatibilities and conflicts across individuals. The first dimension has been called Openness to Change vs. Conservation and contrasts an orientation toward hedonistic goals and pursuit of independent thoughts and actions vs. an endorsement of goals that highlight preserving the social and cultural traditions and order, restraining personal actions not to upset social conventions, and a concern with the security of one's family and country. The second dimension was labeled self-transcendence vs. self-enhancement. This dimension differentiates more prosocial and altruistic concerns with the wellbeing of close and distant others, maintaining fairness within one's society and a concern with the state of the social and natural environment in general from more self-centered concerns that focus on advancing one's power, wealth, and status as well as demonstrating one's abilities and dominance in ways that conform to socially acceptable standards.

**Figure 1:**
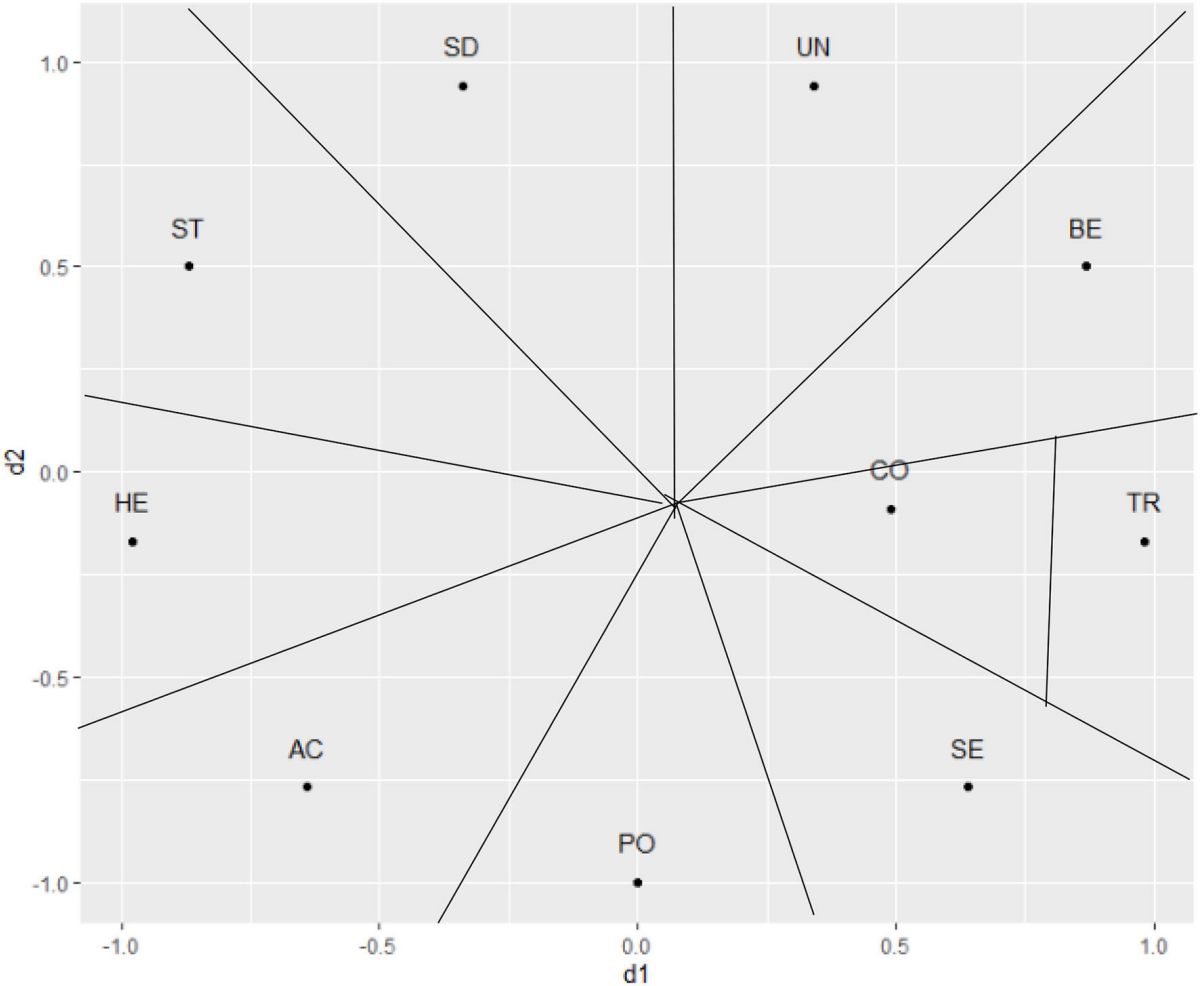
Schematic representation of the Schwartz Value Theory. SD, Self direction; ST, Stimulation; HE, Hedonism; AC, Achievement; PO, Power; SE, Security; TR, Tradition; CO, Conformity; BE, Benevolence; UN, Universalism.

Figure 1 offers a conceptual representation of the two major dimensions and the 10 value types that are encapsulated within them. Finer theoretical distinctions are possible (Schwartz et al., 2012), but here we keep with the more common focus on the 10 value types within the two major dimensions (Fischer, 2017; Schwartz, 2017). Specifically, Benevolence captures the importance of caring about the wellbeing of individuals emotionally close to the person; Universalism captures the appreciation, tolerance, and protection of the wellbeing of all people and nature, independent of the emotional closeness; Conformity values emphasize the restraint of actions or impulses that may upset others or violate social norms and expectations; Tradition values relate to the preservation, respect, and commitment to norms and customs of one's traditional culture or religion; Security concerns the safety and stability of one's close relationships as well as society; Power values emphasize control and dominance of other people and resources; Achievement values focus on demonstrating success and competence according to socially accepted standards; Hedonism values capture the striving for pleasure and gratification of personal desires; Stimulation values highlight excitement, seeking challenges, and novelty in life and finally, Self-Direction values emphasize independence in both thought and action.

This value structure has been transformed into a number of self-report measures (Schwartz, 1992; Schwartz et al., 2001, 2012; Lindeman and Verkasalo, 2005; Lee et al., 2008; Verkasalo et al., 2009; Schwartz and Cieciuch, 2021), which appear to capture these two major dimensions in line with the theory. Demonstrating the conceptual validity, the two major dimensions have been identified using reaction time and neuroscience methodologies (Pakizeh et al., 2007; Brosch et al., 2011; Leszkowicz et al., 2017), and can also be measured in a picture format (Döring et al., 2010).

Predictions based on the theory have so far mostly been tested with data that focus on differences between individuals. On the other hand, a recent set of studies focusing on values as motivational goals within specific situations, that is, value states instead of more trans-situationally stable value traits, has suggested that values expressed within specific situations may relate to other values differently when compared to their relationship across situations and between individuals (Skimina et al., 2021). In other words, previous research has focused on value importance ratings and compared these across individuals, effectively generalizing across situations.

When focusing on specific situations, slightly different associations may emerge because different combinations of values may be salient within a situation to achieve a goal. Individuals are able to pursue two values that are conceptually conflicting in different everyday situations, which may mean that across situations, both values are salient. A good example is the importance of self-enhancement values of achievement within work or academic settings, but the importance of self-transcendence values of benevolence in family settings. In line with the reasoning, Skimina et al. (2021) reported that Power and Hedonism values are positively correlated when examining individual differences across situations as predicted by the theory, but within specific situations, the two value types are not simultaneously activated, leading to an orthogonal relationship between these two value types *in situ*. As we are focusing on narratives, it becomes relevant to ask whether descriptions of specific events may result in different structures. Such distinctions are conceptually important as we describe in the next section.

### Narratives, Autobiographies, and Implicit Values

Understanding a person requires paying attention to different levels or principles of what it means to be a person (McAdams, 1995; McAdams and Pals, 2006). At the most basic level, it is possible to describe individuals as belonging to the human species that emphasizes our communalities. At the most specific level, it is possible to focus on the unique aspects of each person's life. At this level, we can distinguish individual elements of the biography and identity one individual from all other individuals. Despite these differences in abstraction and detail, work on life stories has suggested that these different levels and perspectives are interlinked and integrated. At the lowest level, according to McAdams, are basic biologically driven predispositions. The most widely used descriptions at this level use personality traits, such as the Big Five or the approach vs. avoidance motivational systems (Corr and Cooper, 2016; Soto and John, 2017). At an intermediate level in this framework are aspirations, values, and beliefs that link basic biological predispositions to the wider social and cultural environment. Values are often thought to be operating at this intermediate level. At the most person-specific level, there are rich lived stories that weave together the strands across these different levels, linking basic predispositions to the goals, motivations, and values encountered byan individual in specific situations. From this perspective, autobiographical life stories build on and explicate the individual's choices in the past. These stories are meaningful from a person-focused level of analysis because they provide unity to a person's experience, and provide purpose and meaning.

These stories rely on verbal descriptions of events in a person's life. A person has choices in describing these events, drawing upon a vast vocabulary. The specific choices of words used to describe one's life story can be analyzed and provide insights into the major concerns and preoccupations of the individual. Linguistic choices are behavioral expressions that are worth exploring, an idea that traces back to the linguistic hypothesis first sketched in 1884 by Galton (1949). Such a cognitive approach has been extensively used within the personality domain: language can provide information on what a person attends to, what they associate with objects or events and how they interpret their world, and thereby allowing a careful listener to gain relevant information on the personality of the person (Chung and Pennebaker, 2008).

We propose that the analysis of linguistic choices can provide meaningful information on the values that implicitly guide the individuals. Extracting value information adds an additional layer as values are evaluative standards, that is, they go beyond descriptions of what a person attends to or has done (as typically presumed in personality trait research) and provide insights into how individuals interpreted situations, individuals, and events in their lives. By definition, values are evaluation standards that help individuals make sense of their world (Schwartz and Bilsky, 1987). To the extent that values also reflect motivational goals, extracting information on values can theoretically provide a deeper layer of a person's psychological make-up. By analyzing the choices of words out of a universe of words when describing an event in one's life, it should be possible to derive information about both the evaluative standards and motivations of an individual, that is, their salient values. This is an extension of previous lexical analyses of values of speeches, newspaper texts, and blogs (Bardi et al., 2008; Portman, 2014; Boyd et al., 2015; Ponizovskiy et al., 2020) to the context of autobiographical narratives. We believe that the study of values in autobiographies has much potential because autobiographical narratives have a rich conceptual foundation as a source of personal information (McAdams, 1995).

Our approach shares some similarity with an older tradition focusing on implicit motives (Schultheiss and Pang, 2007). Recent psychometric work in this tradition has also focused on narratives, but in response to standardized ambiguous material to elicit salient motives (McClelland et al., 1989; Schultheiss and Pang, 2007). The basic idea within this research tradition is that the presentation of standardized, but ambiguous stimuli will arouse specific motivational content of importance to the individual and results in a projection of this personally important content onto the generation of the narrative. It is possible to use both content-based coding options (Winter, 2016) and dictionaries (Schultheiss, 2013) to extract salient motives. Although motives are not identical to values, they are conceptually related. Furthermore, an alignment of implicit motives and explicit values appears to increase the wellbeing (Hofer et al., 2006).

This related line on implicit motives offers an interesting additional perspective on lexical analyses of values within narratives. Studying both personally experienced autobiographical stories and stories elicited from ambiguous stimulus materials, these two methods provide an interesting cross-check on our ability of analyzing word choices to index value information about an individual. In summary, we will use both autobiographical life stories and stories produced in response to ambiguous stimuli to examine how well they correlate with explicit self-ratings on value scales.

### Dictionary Approaches to Value Measurement

The focus so far has been on the linguistic choices, that is, the verbal behavior of the actor (Ponizovskiy et al., 2020). These behaviors need to be coded in objective, valid, and reliable ways. Manual coding procedures for value content are available, but are relatively subjective and highly labor-intensive (Suedfeld et al., 2010, 2011; Portman, 2014). A first value dictionary was very brief and included only 3 value terms per value type (Bardi et al., 2008). This will lead to very sparse data matrices when based on naturally occurring language. The most comprehensive dictionary to date has over 1,000 entries and was developed using both theoretical criteria and previous value lists (Ponizovskiy et al., 2020). This list was validated against different types of blogs, text containing fiction from a large American English corpus of texts, Facebook status updates, and short essays in which participants had to describe their values and behavior. The validation was primarily focused on internal relations between the individual value terms. Overall, the higher order motivational domains were well replicated across the five different data sets. An analysis of the position of the 10 value types within a two-dimensional representation suggested greater variability. Notably, universalism values tended to merge with Conservation values and Hedonism and Benevolence values tended to be located quite close to each other, suggesting greater motivational complexity than predicted by Schwartz's model.

A subset of participants from Ponizovskiy et al. (2020) Facebook study and essay study provided self-ratings of their values using the Schwartz Value Survey (Schwartz, 1992). The results showed that 7 out of the 10 correlations between self-reports and value dictionary-derived scores were significant in the essay study and 6 out of the 10 in the Facebook study. The correlations between conceptually matched scores varied between 0.10 and 0.16 in the Facebook study and between 0.12 and 0.31 in the essay study. Maybe this latter finding is not surprising given that the task for the essay was to describe one's values. These analyses therefore suggest it is possible to extract value information with this theory-based value dictionary from texts produced by individuals in different formats and platforms.

To the extent that such analyses are part of an analysis from a distance, it may be interesting to describe the most salient values of an individual or a group. Within self-report measures, there is a relatively consistent hierarchy of values in terms of importance across representative, student, and teacher samples (Schwartz and Bardi, 2001): Benevolence values are typically ranked most important, followed by self-direction values and then universalism values. Similarly, these values and some of the achievement values show the greater consensus within nations and the weakest cross-cultural differences (Fischer and Schwartz, 2011). This suggests that there is a relatively universal ordering of values in terms of their importance in people's life and it would be informative to see whether values extracted in autobiographical or ambiguous stories (which are thought to activate salient motives) show a similar pattern on average.

Across three studies that differed in the content and format of freely generated texts, we aimed to examine how well we can extract personal values from these texts using a pretested theory-driven value dictionary. We addressed the following research questions: First, what are the overall value priorities extracted from texts, and to what extent do they agree with value priorities derived from responses to standard self-rating questionnaires? Second, what is the structure of values derived from texts and to what extent does it correspond to the theoretical model and to the theory-based? Third, to what extent are value patterns consistent across texts generated in different narrative tasks?

## Study 1

Our first study aims to test whether value-relevant information can be extracted from two short autobiographical stories using a recently proposed theory-based value dictionary. This is the first test to check whether value information extracted from two short autobiographical statements using this dictionary correlates with value self-reports. Furthermore, we compare the value importance scores extracted from short autobiographical stories with ordering of self-rated values. We also aim to examine the internal structure of values within autobiographical narratives, that is whether value terms in relation to this specific autobiographical event cluster as expected by the theory.

### Methods

We recruited young adults enrolled in an introductory psychology course in New Zealand. A total of 106 individuals participated. Mean age was 19.6 years (SD = 3.32, Min = 17, Max = 46) and 31 participants (29.2%) were males. Thirteen participants (12.3%) were non-native English speakers. All individuals received partial course credit in return for participating in this study. The study was conducted online in 2019. The study was approved by the School of Psychology Human Ethics Committee under delegated authority of Victoria University of Wellington's Human Ethics Committee (ID0000023640).

#### Procedure

We gave individuals two tasks based on the life narrative writing tasks (Rubin et al., 2009). We always first asked individuals to write about a positive experience: “Think of an episode in your life which you consider a POSITIVE EXPERIENCE, and which has influenced you. Describe in detail WHAT has happened, WHERE, WHEN and WHO was involved. Describe what you THOUGHT or FELT.” We then asked about a challenging episode: Looking back over your whole life so far, select an episode which you would consider a GREAT CHALLENGE that you had to face. Describe in detail WHAT has happened, WHERE, WHEN and WHO was involved. Describe how you ADDRESSED the challenge. Describe what you THOUGHT or FELT.” We always encouraged individuals to first think for a minute before they started writing. We also requested full sentences and text that avoided slang. As a guide, we suggested individuals to write between 20 and 30 lines.

Self-reported values were measured with an adapted gender-neutral version of the Portrait Value Questionnaire (Schwartz et al., 2012). The scale consists of 57 items that describe individuals with specific value priorities. Participants have to respond whether the statements involving various value-driven actions describe them using a scale varying from 1 (Not like me at all) to 6 (Very much like me). Example items were “It is important to me to form my views independently” and “It is important to me that my country is secure and stable.” We used the 10 value types for the present study and calculated Cronbach's alpha using the raw data. Refer to Table 1 for more information. Achievement, Stimulation and Hedonism values had reliabilities below 0.70 [for a discussion of reliability issues with this measure, refer to Schwartz and Cieciuch (2021)]. To examine the theoretical structure, we conducted a multidimensional scaling analysis with the smacof package (Mair et al., 2021) and Euclidean distances based on Pearson's correlations, ordinal data structure, and extracting two dimensions. Compared to the theory-predicted positions (Bilsky et al., 2011), our data showed acceptable conceptual similarity (congruence = 0.88) in line with minimum standards for conceptual replication (Fischer and Fontaine, 2011; Fischer and Karl, 2019). Since there is an ongoing debate in the literature about the need to ipsatize value scores prior to analysis (Fischer, 2004; Rudnev, 2021), we also computed ipsatized values by subtracting the overall value mean from each individual value item and then averaged these ipsatized scores per value type.

**Table 1:** Mean scores for the text-based and rating-based value scores.

	**Raw text**		**Ipsatized text**		**Raw rating**			**Ipsatized rating**	
**Value**	***M***	**SD**	***M***	**SD**	***M***	**SD**	**α**	***M***	**SD**
SD	4.69	2.85	0.15	0.08	4.60	0.82	0.81	0.35	0.57
ST	3.72	2.68	0.12	0.08	4.32	0.86	0.63	0.06	0.71
HE	2.22	1.91	0.08	0.07	4.81	0.81	0.67	0.55	0.64
AC	5.64	4.81	0.19	0.13	4.52	0.84	0.58	0.26	0.62
PO	1.40	1.45	0.05	0.05	3.09	0.87	0.75	−1.16	0.90
SE	1.02	1.31	0.03	0.04	4.25	0.79	0.75	0.00	0.47
TR	0.52	1.73	0.02	0.04	3.10	1.27	0.78	−1.15	1.20
CO	1.79	0.94	0.07	0.04	4.01	0.75	0.75	−0.24	0.52
BE	7.63	4.72	0.25	0.12	4.91	0.79	0.84	0.66	0.53
UN	1.90	2.00	0.06	0.06	4.72	0.80	0.87	0.47	0.56

*SD, Self direction; ST, Stimulation; HE, Hedonism; AC, Achievement; PO, Power; SE, Security; TR, Tradition; CO, Conformity; BE, Benevolence; UN, Universalism*.

#### Dictionary

We used the refined value dictionary developed by Ponizovskiy et al. (2020). The refined value dictionary contains theory-driven selections of adjectives, verbs, and nouns that have been shown to correlate with value priorities across a range of textual sources. The refined dictionary contains 1,068 entries. The values are not equally represented in the dictionary: the smallest word set captures Security values (85 words) and the largest word set is the Self-direction value list (140 words; Ponizovskiy et al., 2020).

#### Data Treatment

To facilitate analyses, we first converted the texts into a format that facilitates subsequent analyses, using tokenization and part-of-speech (PoS) tagging from the R version of spaCy (Benoit et al., 2020), a wrapper around the natural language processing library in Python (Van Rossum and Drake, 2011). Tokenization automatically splits the texts into single word units, and PoS tagging annotated each word with word type, such as noun, adjectives, and verbs. We then matched words in each person's text with the dictionary. If a person used a word from the dictionary, this was counted as a hit. All hits were summed per dictionary value category for each person. No spelling correction or lemmatization was used. Similar to value ratings, it is possible to use an adjusted value score which adjusts the frequency for each value type for the overall number of value terms used by the participant. Therefore, this score (which we refer to as *ipsatized* text-based value score henceforth) could be interpreted similarly to ipsatized rating scores. It is also possible to compute an adjusted score as reflecting the overall text length (dividing each text-based value score by the overall text length). We do not report these scores here, because the results were qualitatively very similar to the ipsatized scores, which are more commonly reported in the value literature. All analyses are based on Pearson's product moment correlations.

To examine the overall content of both writing prompts, we fitted a bi-term topic model (Yan et al., 2013) using the BTM package (Wijffels, 2021a) in R on each separate text. Initially, we used UDPipe (Wijffels, 2021b) to tokenize and annotate the text for each participant. For the analysis, we focused on the lemmas of verbs, nouns, and adjectives. To achieve a robust estimation of term -topic relationship, we repeated the Gibbs sampling process 1,000 times allowing the extraction of up to eight topics. The graphical representations of the extracted topics are available in the Supplementary Material.

### Results

First, we examined basic descriptive information. On average, participants produced 490 words (SD = 4.25; min = 190, max = 1,150). This is above the average word count in the essay study in the original validation study of the value dictionary (206 words), and our lower limit is aligned with the lower limit of 200 words set in the Facebook validation study (Ponizovskiy et al., 2020).

Second, on average, participants used 30.52 words from the value dictionary (SD = 10.4, min = 8, max = 67, median = 28.0). This means that on average, 6.38% of the terms used were entries in value dictionary used (SD = 1.69, min = 2.34, max = 11.34, and median = 6.21). This suggests that value terms are relatively common in autobiographical stories.

Third, focusing on the overall value profiles, the most important value type using the rating scale was Benevolence, followed by Hedonism and Universalism (using both raw and ipsatized scores). When using the text-based value scores, the most important values were Benevolence, followed by Achievement and Self-Direction (for both raw scores and ipsatized scores). The least important value types in ratings were Power, Tradition, and then Conformity. For the text-based value scores, the least important value types were Tradition, Security, and then Power. This suggests that most and least important values differed somewhat across rating vs. text-based analyses. The correlation of the value profiles with the raw data was 0.62.

To examine the similarity in greater detail at the level of individual differences, we next analyzed the similarity between the self-reports and the text-based value scores. Table 2 shows the summary of the motivation-matched correlations and Table 3 shows the full correlation table across methods. A few results are noteworthy. First, the average correlations suggested the strongest average relations for the raw text scores with the raw rating scores (mean *r* = 0.07). The next strongest average correlation was between ipsatized rating scores and raw text-based scores (mean *r* = 0.04). There have been strong arguments for and against ipsatization to strengthen value profiles (Schwartz, 1992; Schwartz et al., 2001; Fischer, 2004; He and van de Vijver, 2015; Rudnev, 2021); our data showed that raw scores matched with motivational content for both self-ratings and text-based data correlated on average slightly stronger (Δ*r* = 0.03). Second, these average correlations were similar to the average correlation between text-based and rating-based value scores in Ponizovskiy et al. (2020) when using Facebook status updates as input (mean correlation was 0.07). The average correlation for texts in which students had to describe their values was higher (mean *r* = 0.16), as might be expected. Therefore, our mean correlations were weak, but in line with previous research that used text that is not explicitly value focused. Third, the highest correlations for matching value types were observed for Tradition (*r* = 0.16), followed by Conformity (*r* = 0.15) and Power (*r* = 0.13), when using the raw scores for both types of variables. The pattern was identical when examining the correlations between raw ratings and adjusted scores using text-based values. When using ipsatized ratings, the associations were somewhat different. For ipsatized ratings with text-based scores, the strongest correlation now was observed for Hedonism (*r* = 0.24 and 0.08, for raw and ipsatized text-based scores, respectively), followed by Power (*r* = 0.12 and 0.11, for raw and ipsatized text-based scores, respectively) and Tradition (*r* = 0.07 and 0.10 for raw and ipsatized text-based scores, respectively). Hence, Tradition and Power showed relatively consistent positive correlations, irrespective of the specific type of score adjustment used. A final observation is that the off-diagonal elements in a majority of cases were of similar or larger absolute value, suggesting that the text-based value scores correlated more strongly with other value types rather than the intended value type. In some cases, these larger values were found with adjacent values, in some cases with motivationally opposing values (which is appropriate as long as the correlation is negative, indicating that motivationally conflicting values are negatively correlated with each other), but a considerable number of absolute correlations on the off-diagonal were larger for motivationally incongruent values. In other words, no clear pattern indicating discriminant validity was observable.

**Table 2:** Overall similarity (correlation) of matching value types in Study 1.

	**Raw text × raw rating**	**Ipsatized text × raw rating**	**Raw text × ipsatized** **rating**	**Ipsatized text × ipsatized** **rating**
SD	0.00	−0.04	−0.02	0.01
ST	0.00	−0.04	0.02	−0.02
HE	0.06	−0.09	0.24*	0.08
AC	−0.07	−0.06	−0.06	−0.04
PO	0.13	0.14	0.12	0.11
SE	0.05	0.08	−0.07	−0.08
TR	0.16^#^	0.15	0.07	0.10
CO	0.15	0.10	0.05	0.02
BE	0.09	0.04	−0.01	−0.03
UN	0.09	0.02	0.06	0.04
Mean	0.07	0.03	0.04	0.02

^#^p < 0.10, *p < 0.05.

*SD, Self direction; ST, Stimulation; HE, Hedonism; AC, Achievement; PO, Power; SE, Security; TR, Tradition; CO, Conformity; BE, Benevolence; UN, Universalism*.

**Table 3:** Correlation matrix across types of score adjustment in Study 1.

		**Raw ratings**										**Ipsatized rating**									
		**SD**	**ST**	**HE**	**AC**	**PO**	**SE**	**TR**	**CO**	**BE**	**UN**	**SD**	**ST**	**HE**	**AC**	**PO**	**SE**	**TR**	**CO**	**BE**	**UN**
		**Ratings**																			
Raw text	SD	0.00	0.09	0.04	−0.07	−0.01	0.00	0.03	0.04	−0.01	−0.03	−0.02	0.09	0.03	−0.11	−0.02	−0.02	0.03	0.04	−0.04	−0.06
	ST	−0.14	0.00	−0.01	−0.12	0.12	−0.06	−0.07	0.00	−0.01	0.01	−0.19	0.02	0.01	−0.15	0.12	−0.08	−0.07	0.02	0.00	0.04
	HE	−0.10	−0.17	0.06	−0.12	−0.18	−0.22	−0.01	−0.14	−0.16	−0.14	0.05	−0.05	0.24	0.01	−0.06	−0.14	0.08	0.01	−0.03	−0.01
	AC	−0.03	−0.03	−0.09	−0.07	−0.13	−0.07	0.01	−0.01	0.06	0.03	0.00	0.00	−0.08	−0.06	−0.10	−0.07	0.03	0.03	0.14	0.08
	PO	−0.16	0.08	0.09	0.01	0.13	−0.02	−0.04	0.03	−0.10	0.01	−0.24	0.09	0.11	0.01	0.12	−0.05	−0.05	0.03	−0.15	0.00
	SE	0.15	0.24	0.18	0.12	−0.03	0.05	−0.02	0.01	0.11	0.17	0.09	0.18	0.12	0.05	−0.11	−0.07	−0.08	−0.13	0.02	0.10
	TR	0.09	0.18	0.07	0.07	0.11	0.16	0.16	0.26	0.10	0.12	−0.08	0.04	−0.09	−0.09	−0.02	0.01	0.07	0.14	−0.08	−0.04
	CO	0.00	0.12	0.12	0.09	−0.04	0.04	0.01	0.15	0.15	0.24	−0.15	0.03	0.02	−0.01	−0.14	−0.12	−0.06	0.05	0.06	0.18
	BE	0.11	0.03	0.18	0.09	−0.03	0.20	0.13	0.09	0.09	0.05	0.03	−0.07	0.11	0.01	−0.12	0.18	0.08	−0.02	−0.01	−0.07
	UN	0.06	0.06	0.19	0.06	−0.09	0.07	−0.14	0.09	0.04	0.09	0.02	0.02	0.19	0.02	−0.12	0.05	−0.17	0.05	0.00	0.06
Ipsatized text	SD	−0.04	0.06	−0.04	−0.08	0.01	−0.09	−0.03	−0.03	−0.07	−0.10	0.01	0.13	0.00	−0.05	0.05	−0.07	0.00	0.03	−0.03	−0.08
	ST	−0.15	−0.04	−0.04	−0.08	0.18	−0.07	−0.07	−0.05	−0.01	−0.04	−0.18	−0.02	−0.02	−0.07	0.20	−0.07	−0.05	−0.03	0.02	−0.02
	HE	−0.08	−0.21	−0.09	−0.15	−0.19	−0.23	0.02	−0.14	−0.17	−0.15	0.10	−0.08	0.08	0.00	−0.04	−0.13	0.12	0.03	−0.03	0.00
	AC	0.01	−0.03	−0.09	−0.06	−0.13	−0.06	−0.03	−0.04	0.04	0.04	0.07	0.00	−0.06	−0.04	−0.09	−0.05	−0.01	0.00	0.12	0.11
	PO	−0.10	0.06	0.10	0.07	0.14	0.00	−0.09	0.03	−0.05	0.04	−0.18	0.04	0.10	0.06	0.11	−0.03	−0.11	0.01	−0.12	0.02
	SE	0.19	0.28	0.17	0.17	0.01	0.08	0.01	0.06	0.15	0.18	0.10	0.20	0.06	0.08	−0.10	−0.08	−0.08	−0.11	0.04	0.09
	TR	−0.01	0.10	−0.05	0.04	0.16	0.15	0.15	0.20	0.04	0.05	−0.16	0.01	−0.19	−0.07	0.06	0.08	0.10	0.14	−0.10	−0.07
	CO	0.06	0.10	−0.07	0.09	0.05	0.09	0.06	0.10	0.06	0.14	−0.03	0.02	−0.19	0.01	−0.03	0.00	0.01	0.02	−0.04	0.09
	BE	0.10	−0.06	0.08	0.11	0.04	0.19	0.14	0.04	0.04	−0.02	0.05	−0.14	0.02	0.06	−0.02	0.22	0.11	−0.04	−0.03	−0.12
	UN	0.03	0.01	0.14	0.04	−0.08	0.02	−0.20	0.00	0.01	0.02	0.05	0.02	0.19	0.06	−0.07	0.04	−0.21	0.01	0.03	0.04

*Values above r = 0.19 are significant at p < 0.05*.

*SD, Self direction; ST, Stimulation; HE, Hedonism; AC, Achievement; PO, Power; SE, Security; TR, Tradition; CO, Conformity; BE, Benevolence; UN, Universalism*.

*The meaning of the color indicates the shaded entries show value-type matched correlations*.

To provide a more holistic examination of the similarity of the correlation matrices, we computed Mantel correlations to examine the overall similarity of the matrices (refer to Table 4). As can be seen there, the raw rating correlation matrix and the raw text-based value dictionary matrix showed a significant and positive correlation, compared to the adjusted scores. Therefore, the raw data-based correlation matrices showed some overall and significant similarity in the correlation patterns.

**Table 4:** Overall similarity of the correlation matrices (using Mantel test) in Study 1.

	**Raw text**	**Ipsatized text**	**Raw rating**
Raw text			
Ipsatized text	0.46**		
Raw ratings	0.33*	−0.19	
Ipsatized ratings	0.16	−0.15	0.76**

^**^p < 0.001, ^*^p < 0.05.

**Figure 2:**
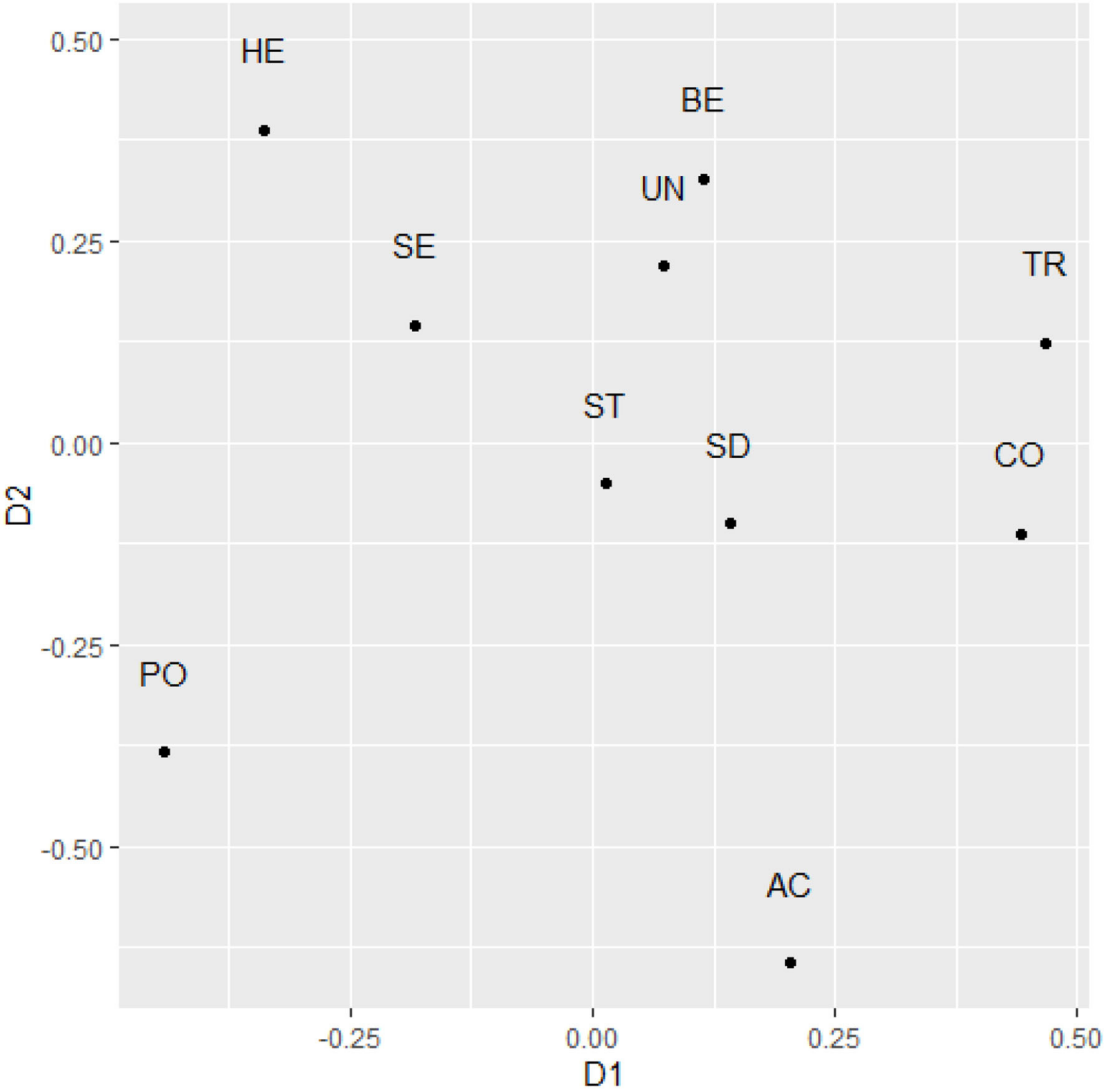
The two-dimensional representation of raw text-based value scores after rotation to maximal similarity with theoretical predictions in Study 1. SD, Self direction; ST, Stimulation; HE, Hedonism; AC, Achievement; PO, Power; SE, Security; TR, Tradition; CO, Conformity; BE, Benevolence; UN, Universalism.

Finally, we conducted a multidimensional scaling analysis of the raw data of text-based scores, using the smacof package (Mair et al., 2021). We specified ordinal data structure and plotted two dimensions. Stress 1 for this two-dimensional solution was 0.13, which is comparable to previous MDS stress values. In order to interpret the matrix, it is necessary to rotate the empirical structure to similarity with the expected structure. We used the theory-predicted positions as the target matrix (Bilsky et al., 2011). Compared to ideal theory-based matrix, the congruence coefficient of the solution was 0.878 (alienation coefficient: 0.479), suggesting a conceptual similarity in structure (Fischer and Fontaine, 2011; Fischer and Karl, 2019). When focusing on the individual dimensions, the estimates of the individual dimensions suggested lower replicability; for dimension 1, we found *r* = 0.41 and for dimension 2, *r* = 0.46. Examining the position of the individual value types (Figure 2), achievement values were positioned in between power values toward the conservative value end (positive end of dimension 1). Achievement and Power were located nearly at opposite ends of dimension 1, which suggests lower motivational congruence based on the extracted value scores in these autobiographical stories. Security values emerged close to Hedonism values, which is theoretically unexpected. Stimulation and self-direction values emerged in the center of the two-dimensional representation. In line with the theory, tradition and conformity values emerged close together, similar to benevolence and universalism values.

### Discussion

Our first exploration of the value-based scores in two short autobiographical stories suggested mixed evidence. On the one hand, the average correlation was similar to the correlations observed in Facebook updates reported in the original validation study (Ponizovskiy et al., 2020), which is encouraging. The raw data matrices also showed some significant similarity overall and the individual value correlations suggested comparable patterns to previous studies. The structure showed reasonable similarity with the overall expected structure, even though individual dimensions were not all positioned as expected by theory. On the other hand, the salient values in this sample diverged between the rating and text-based scores, although the profiles were somewhat similar. The correlation patterns showed that there were relatively low levels of convergent and discriminant validity.

One interesting observation was the emergence of achievement values as one of the most salient values in the autobiographical narratives. Participants in this study wrote about a positive and a challenging experience. It seems that these achievement values are among the salient values that occur more when individuals describe and reflect on these events, which appear valid as challenges often involving the need for overcoming obstacles that show performance according to social standards (achievement values according to Schwartz's definition). Hence, the divergence of the value priorities between self-ratings and values extracted from these narratives may be explicable by the nature of the autobiographical memories that individuals were asked to share.

Of relevance to value researchers are the ipsatized scores that did not improve the patterns in line with theory. When examining the overall patterns *via* Mantel correlations, the ipsatized matrices showed qualitatively divergent patterns of correlations between ratings and text-based scores. Given the debates about the interpretation of adjusted scores and their relative performance compared to the raw data in the current data, it seems more prudent to work with raw scores, both for ratings and for the text-based value scores, because raw scores do not entail the same assumptions as adjusted scores.

However, the first study was based on a sample of 106 individuals which was asked to describe only two autobiographical stories. These two stories were also particularly focused on emotionally salient events, which may not necessarily strongly activate values. It would be useful to extend these tasks and examine whether a stronger pattern can be observed when asking individuals to provide autobiographical stories at a broader level.

## Study 2

This study was intended to replicate and extend the first study. First of all, we recruited a larger sample and asked participants to report a larger number of autobiographical stories. Instead of focusing individuals on stories with a specific emotional content, we asked for a larger number of general autobiographical memories that are salient for individuals. Therefore, the goal of the second study was to extend Study 1 by using a more elaborate autobiographical narrative task.

### Methods

We recruited young adults enrolled in an introductory psychology course in New Zealand. A total of 152 individuals (of which 111 were females, 73.0%) participated, with a mean age of 19.51 years (SD = 3.91, min = 17, max = 41). Twenty participants (13.2%) were non-native English speakers. All individuals received partial course credit in return for participating in this study. The study was conducted online in 2020 and was approved by the Victoria University of Wellington Human Ethics Committee (RMP0000028177).

#### Autobiographical Writing Tasks

We asked individuals to describe central events in their lives (adapted from the life narrative writing tasks; Rubin et al., 2009). The specific instructions were as follows: “This part deals with your personal life story. Your task is to decide which events are most central to the story of your own life. It has to be events that you have personally experienced. It is your personal life and personal life story that this task is about. There are no right or wrong answers. You are the one who knows best what has been central to your life. Imagine that you are to tell your life story to a new friend, whom you have just met and who therefore doesn't know anything about your past. It is a (fictitious) friend with whom you are absolutely confident and with whom you can be completely honest. Your task is to note the seven memories about events from your own personal life that you think are most central to your life story. Describe in detail WHAT has happened, WHERE, WHEN and WHO was involved. Describe what you THOUGHT or FELT. Think for a minute before you start writing. Write between 20 and 30 lines.”

#### Value Measures

We used the same value instrument as in Study 1. Reliabilities are shown in Table 5. Only Achievement values had internal consistencies below 0.70. An MDS analysis showed acceptable replicability in relation to the theoretically proposed structure (congruence = 0.92).

**Table 5:** Overall value means for different data types and score adjustments in Study 2.

	**Raw text**		**Ipsatized text**		**Raw rating**			**Ipsatized rating**	
	***M***	**SD**	***M***	**SD**	***M***	**SD**	**α**	***M***	**SD**
SD	16.23	10.91	0.17	0.07	4.89	0.66	0.79	0.47	0.50
ST	6.95	5.05	0.08	0.05	4.44	0.93	0.71	0.02	0.80
HE	9.68	7.18	0.11	0.07	5.03	0.86	0.79	0.61	0.71
AC	11.38	8.36	0.12	0.07	4.47	0.76	0.50	0.05	0.64
PO	4.18	3.53	0.05	0.05	2.76	0.93	0.84	−1.66	0.93
SE	4.27	3.34	0.05	0.05	4.51	0.73	0.75	0.10	0.48
TR	2.71	8.66	0.02	0.05	3.24	1.30	0.84	−1.18	1.18
CO	2.93	2.61	0.03	0.03	4.37	0.72	0.78	−0.05	0.53
BE	28.48	16.03	0.31	0.11	5.22	0.61	0.77	0.80	0.49
UN	5.30	5.12	0.05	0.04	4.96	0.68	0.85	0.55	0.58

*SD, Self direction; ST, Stimulation; HE, Hedonism; AC, Achievement; PO, Power; SE, Security; TR, Tradition; CO, Conformity; BE, Benevolence; UN, Universalism*.

#### Data Treatment

We used the same data pre-processing as in Study 1. Bi-term representations of the topics generated are available in the Supplementary Material.

### Results

On average, participants produced 1,999.28 words (SD = 984.46; min = 135, max = 7,177). The average number of words included in the value dictionary was 92.1 (SD = 44.7, min = 5, max = 224). Expressed as percentages, on average, 4.62% of the words used in the autobiographies were terms included in the value dictionary (SD = 1.19, min = 1.58, max = 9.12).

In this sample, the most central values from the autobiographical narratives (both raw and value-adjusted) were Benevolence values, followed by Self-direction values and then Achievement values (refer to Table 5). Therefore, the most salient values were quite comparable to Study 1. For the ratings, the most central values in this sample were Benevolence values, followed by Hedonism and then Universalism values. Therefore, the same three values were again the most salient values in this study as in Study 1 when using rating scores. The least salient values were Tradition, Conformity, and Power values for the text-based scores and Power, Tradition, and Security values in the ratings. Overall, the relative pattern again diverged somewhat between the two types of data but was rather similar to Study 1. The correlation of the raw data value profiles was 0.57.

Focusing on the correlations between matched value types (Table 6), the average correlation with raw data was *r* = 0.12; correlations were weaker when comparing adjusted and ipsatized scores. Again, this was comparable to the validation information in Ponizovskiy et al. (2020) study. The strongest correlation in this sample using raw scores was for Universalism values, followed by Stimulation and Achievement values. Universalism also remained the strongest correlation in any of the other combinations, but the second and third strongest correlations varied across combinations of scores compared. When examining the individual patterns of correlations (see Table 7), the pattern appeared somewhat clearer when examining the correlations based on raw data. Three values showed the strongest absolute correlations on the diagonal, and four values showed stronger off-diagonal absolute correlations with motivationally congruent values (or incongruent value types in an expected directions). Text-based Security and Conformity values showed non-congruent correlation profiles with self-ratings.

**Table 6:** The correlations for motivationally matched value types in Study 2.

	**Raw text × raw rating**	**Raw text × ipsatized rating**	**Ipsatized text × raw rating**	**Ipsatized text × ipsatized rating**
SD	0.11	0.12	−0.10	−0.01
ST	0.25**	0.22**	0.13^#^	0.13^#^
HE	0.09	0.09	0.05	0.09
AC	0.23**	0.14^#^	0.21**	0.13
PO	0.13^#^	0.15^#^	0.12	0.17^#^
SE	−0.02	0.01	−0.04	0.04
TR	0.08	0.07	0.10	0.09
CO	−0.02	−0.06	−0.05	−0.05
BE	0.14^#^	0.09	0.09	0.11
UN	0.25**	0.21**	0.25**	0.25**
Mean	0.12	0.10	0.08	0.09

^#^p < 0.10, **p < 0.01.

*SD, Self direction; ST, Stimulation; HE, Hedonism; AC, Achievement; PO, Power; SE, Security; TR, Tradition; CO, Conformity; BE, Benevolence; UN, Universalism*.

**Table 7:** Overall correlation matrix comparing values across data modalities in Study 2.

		**Raw rating**										**Ipsatized rating**									
		**SD**	**ST**	**HE**	**AC**	**PO**	**SE**	**TR**	**CO**	**BE**	**UN**	**SD**	**ST**	**HE**	**AC**	**PO**	**SE**	**TR**	**CO**	**BE**	**UN**
Raw text	SD	0.11	0.05	−0.06	−0.01	0.04	−0.03	−0.12	0.01	0.08	0.03	0.12	0.04	−0.09	−0.03	0.02	−0.07	−0.15	−0.01	0.08	0.02
	ST	0.13	0.25	0.19	0.10	−0.05	−0.01	−0.05	0.06	0.11	0.11	0.06	0.22	0.15	0.03	−0.11	−0.13	−0.10	−0.02	0.03	0.04
	HE	0.03	0.10	0.09	0.03	0.06	−0.01	0.01	−0.02	0.03	−0.01	0.02	0.10	0.09	0.02	0.04	−0.05	0.00	−0.05	0.00	−0.04
	AC	0.19	0.20	0.27	0.23	0.18	0.15	0.06	0.00	0.09	0.01	0.07	0.12	0.20	0.14	0.09	0.04	−0.01	−0.17	−0.07	−0.14
	PO	0.05	0.04	0.01	−0.05	0.13	−0.10	−0.04	−0.09	−0.19	−0.04	0.11	0.07	0.04	−0.03	0.15	−0.11	−0.03	−0.09	−0.19	−0.02
	SE	0.00	−0.12	−0.14	−0.09	−0.15	−0.02	−0.08	0.03	0.04	0.11	0.05	−0.11	−0.14	−0.08	−0.12	0.01	−0.07	0.08	0.10	0.17
	TR	0.03	0.01	0.01	−0.02	0.01	0.06	0.08	0.03	0.06	0.01	−0.01	−0.02	−0.03	−0.06	−0.02	0.03	0.07	−0.01	0.02	−0.03
	CO	0.17	−0.08	−0.05	0.04	0.09	0.03	−0.04	−0.02	−0.04	0.01	0.19	−0.11	−0.09	0.02	0.07	0.01	−0.06	−0.06	−0.09	−0.02
	BE	0.16	0.12	0.09	0.05	0.01	0.02	−0.09	0.04	0.14	0.05	0.13	0.09	0.05	0.00	−0.04	−0.04	−0.13	−0.03	0.09	−0.01
	UN	0.12	0.05	0.03	0.03	0.06	−0.02	−0.04	0.04	−0.03	0.25	0.06	0.00	−0.03	−0.04	0.01	−0.13	−0.09	−0.04	−0.13	0.21
Ipsatized text	SD	−0.10	−0.08	−0.21	−0.08	−0.02	−0.10	−0.15	−0.04	−0.03	−0.08	−0.01	−0.01	−0.17	−0.01	0.04	−0.02	−0.11	0.05	0.09	0.01
	ST	0.01	0.13	0.10	0.05	−0.07	−0.04	−0.03	0.07	0.04	0.06	−0.02	0.13	0.09	0.03	−0.09	−0.10	−0.05	0.06	0.01	0.04
	HE	−0.09	0.04	0.05	−0.03	−0.01	−0.04	0.06	−0.02	−0.04	−0.07	−0.08	0.07	0.09	−0.01	0.01	−0.02	0.08	0.01	−0.01	−0.05
	AC	0.11	0.15	0.26	0.21	0.14	0.16	0.10	0.01	0.04	−0.02	−0.01	0.08	0.21	0.13	0.06	0.09	0.05	−0.12	−0.11	−0.15
	PO	−0.06	−0.06	−0.07	−0.08	0.12	−0.10	0.02	−0.10	−0.24	−0.10	0.01	−0.01	−0.02	−0.02	0.17	−0.05	0.07	−0.05	−0.21	−0.04
	SE	−0.09	−0.16	−0.19	−0.10	−0.15	−0.04	−0.01	−0.01	0.01	0.06	−0.03	−0.13	−0.17	−0.05	−0.10	0.04	0.02	0.07	0.10	0.15
	TR	0.03	−0.01	−0.01	−0.05	−0.01	0.08	0.10	0.03	0.05	0.02	−0.01	−0.05	−0.04	−0.09	−0.03	0.07	0.09	0.00	0.02	−0.02
	CO	0.08	−0.15	−0.11	−0.01	0.09	0.03	0.01	−0.05	−0.10	−0.06	0.13	−0.16	−0.12	0.00	0.11	0.07	0.02	−0.05	−0.10	−0.06
	BE	0.02	0.01	0.04	0.01	−0.06	0.00	−0.03	0.03	0.09	0.00	0.02	0.01	0.04	0.00	−0.07	0.00	−0.03	0.04	0.11	−0.01
	UN	0.10	−0.03	−0.02	0.01	0.03	−0.03	−0.06	0.02	−0.05	0.25	0.07	−0.08	−0.07	−0.04	0.00	−0.11	−0.10	−0.04	−0.13	0.25

*Correlations above r = 0.16 are significant at p < 0.05*.

*SD, Self direction; ST, Stimulation; HE, Hedonism; AC, Achievement; PO, Power; SE, Security; TR, Tradition; CO, Conformity; BE, Benevolence; UN, Universalism*.

*The meaning of the color indicates the shaded entries show value-type matched correlations*.

However, when examining the similarity of the value correlation matrices, the overall similarity was substantively lower and not significant (*r* = 0.14, Table 8).

**Table 8:** Overall similarity of the correlation matrices (using Mantel correlations) in Study 2.

	**Raw text**	**Ipsatized text**	**Raw rating**
Raw text			
Ipsatized text	0.03		
Raw rating	0.14	−0.06	
Ipsatized rating	0.12	−0.01	0.80**

The meaning of **symbol indicates the value of p < 0.01.

**Figure 3:**
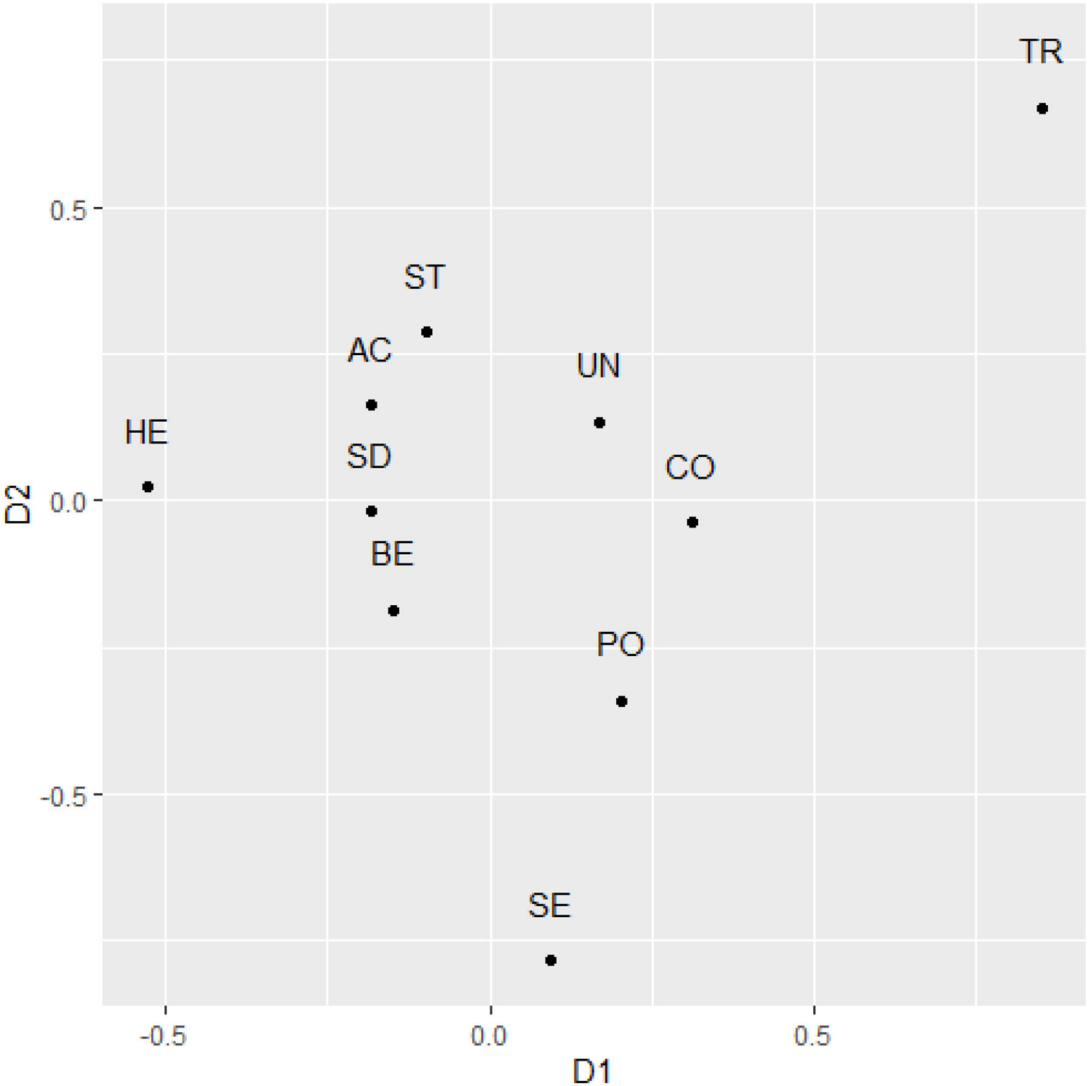
The two-dimensional representation of raw text-based value scores after rotation to maximal similarity with theoretical predictions in Study 2. SD, Self direction; ST, Stimulation; HE, Hedonism; AC, Achievement; PO, Power; SE, Security; TR, Tradition; CO, Conformity; BE, Benevolence; UN, Universalism.

Finally, we examined the structural properties with Multidimensional Scaling, with the same specifications as in Study 1. The value of stress 1 was 0.11. Comparing the structure with theoretically expected coordinates, the congruence coefficient was 0.840 (alienation coefficient 0.542), which suggests quite high replicability. When examining the rotated dimensions, the similarity for dimension 1 was *r* = 0.71, whereas for dimension it was *r* = 0.35. A visual inspection of the rotated matrix suggests that tradition and security values were located at opposing ends of dimension 2 (see Figure 3). Power and Achievement values again show somewhat low compatibility. Power values were also quite closely positioned to the motivationally conflicting Benevolence values.

### Discussion

Our second study expanded the coverage of autobiographical stories. First of all, the most and least salient values in this extended autobiographical task again matched the corresponding values observed in the previous study. Both Self-direction and Achievement values may be salient for individuals when narrating autobiographical stories. In contrast, self-rated values diverged from text-derived scores in terms of the most salient values, emphasizing both more universalistic Other-oriented values as well as Hedonistic values. Both might be expected when asking young adults at a University about their most salient values currently.

Correlations of values matched for content type showed some sizable correlations for this type of data. At the same time, the convergence of overall correlation patterns (including diagonal and off-diagonal elements) for self-rated and text-based value scores was lower.

In the next study, we tested whether a more traditional implicit motivation text might show greater convergence with self-rated values. Such narrative tasks have a long tradition in psychological analyses and have been argued to activate salient motives of an individual (Schultheiss and Pang, 2007). Hence, it would be informative to examine whether it is possible to extract salient values using the value dictionary and then compare the patterns with autobiographical narratives.

## Study 3

The final study focused on a variation of the Thematic Apperception Test, but based on modern psychometric criteria (Schultheiss and Pang, 2007). These tests have a long tradition in psychology, but to the best of our knowledge, no study has been published which aimed to extract value data within a theory-driven value dictionary from these stories and then compare both the relative importance and correlations with self-reports value. Therefore, the goal of our final study was to test dictionary-based value score extractions with a narrative method that has been argued to elicit more implicitly salient motives and orientations.

### Methods

#### Sample

We recruited 150 young adults enrolled in an introductory psychology course in New Zealand. Unfortunately, due to an administrative oversight, no demographic information was collected. All individuals received partial course credit in return for participating in this study. The study was conducted online in 2018 and was approved by the School of Psychology Human Ethics Committee under the delegated authority of Victoria University of Wellington's Human Ethics Committee (ID0000023640).

#### Narrative Task

We used an adapted version of the Telling a story task, which is modeled on the Thematic Apperception Test (Murray, 1943). Participants were presented with four pictures of the “Picture Story Exercise” (Schultheiss and Pang, 2007: boxer, women in laboratory, couple by river, nightclub scene) and were asked to write a complete story about each picture—a story with a beginning, a middle, and an end. Specific focus was given to the description of people in each story, asking participants to try to portray who the people in each picture might be, what they are feeling, thinking, and wishing for. Individuals were instructed to look at the picture for a couple of seconds first and then write whatever story came to their mind. Individuals had 5 min for each story.

#### Value Measures

We used the same value instrument as in Study 1. Reliabilities are shown in Table 9. Only Achievement and Stimulation values showed internal consistencies below 0.70. An MDS analysis suggested satisfactory replication of the theoretically proposed structure (congruence = 0.92).

**Table 9:** Overall mean ratings across data modalities in Study 3.

	**Raw text**		**Ipsatized text**		**Raw rating**			**Ipsatized rating**	
	***M***	**SD**	***M***	**SD**	***M***	**SD**	**α**	***M***	**SD**
SD	2.40	1.93	0.15	0.11	4.70	0.82	0.88	0.48	0.59
ST	1.49	1.54	0.10	0.10	4.32	0.89	0.66	0.09	0.74
HE	1.95	1.63	0.14	0.13	4.72	0.90	0.82	0.49	0.72
AC	3.21	2.61	0.18	0.11	4.44	0.87	0.64	0.21	0.60
PO	1.74	1.91	0.10	0.11	2.98	0.92	0.80	−1.24	0.87
SE	0.83	1.12	0.05	0.08	4.26	0.86	0.82	0.03	0.56
TR	0.33	0.81	0.02	0.04	3.22	1.24	0.82	−1.00	1.13
CO	0.34	0.61	0.02	0.05	3.92	0.98	0.85	−0.30	0.74
BE	3.29	2.95	0.19	0.13	4.93	0.78	0.84	0.71	0.48
UN	0.86	1.09	0.05	0.06	4.56	0.84	0.87	0.34	0.59

*SD, Self direction; ST, Stimulation; HE, Hedonism; AC, Achievement; PO, Power; SE, Security; TR, Tradition; CO, Conformity; BE, Benevolence; UN, Universalism*.

#### Data Treatment

We used the same data pre-processing as in Studies 1 and 2.

### Results

Examining the number of words produced, on average, individuals wrote 303.6 words (SD = 151, min = 42, max = 1,238) and used on average 16.43 value terms (SD = 8.1, min = 1, max = 48). This suggests that about 5.71% of the words mentioned were included in the value dictionary (SD = 2.08, min = 0.32, max = 12.98).

Focusing on the relative importance of individual value types within our sample (Table 9), the most salient values in ambiguous stories were Benevolence values, followed by Achievement and Self-Direction values. This again matches the most important values in the autobiographical stories. The least important values were Conformity, Tradition, and Security. Surprisingly, Universalism values were also very low in importance (coming in a close fourth in terms of least important values). In this sample, the most important self-rated values were Benevolence, Hedonism, and Self-Direction values, again in line with the previous studies. The least important values in the self-ratings were Power, Tradition, and Conformity values. The correlation of the value profiles was 0.52.

When examining the correlations between motivationally matched value types (Table 10), the average correlation was practically zero (*r* = 0.01). The strongest correlation was observed for Benevolence values, followed by Achievement values. Importantly, we observed five negative correlations. This suggests that important values within ambiguous stories are negatively related to self-rated values (Table 10). Concerning the pattern of diagonal vs., off-diagonal correlations (Table 11), only Benevolence values showed the strongest corelation along the diagonal, rather than off-diagonal (with the raw data). A few values showed stronger correlations with motivationally congruent values (e.g., Self-direction and Stimulation values) which is consistent with the value theory, but overall, there was no clearly discernible pattern.

**Table 10:** Correlations between motivationally matched value types in Study 3.

	**Raw text × raw rating**	**Raw text × ipsatized rating**	**Ipsatized text × raw rating**	**Ipsatized text × ipsatized rating**
SD	0.02	0.02	0.05	0.04
ST	0.02	−0.01	0.07	0.07
HE	−0.06	−0.05	−0.02	−0.06
AC	0.07	0.01	0.05	−0.04
PO	−0.01	0.03	0.01	0.09
SE	−0.03	−0.03	−0.05	−0.01
TR	0.04	0.03	0.10	0.09
CO	−0.03	0.01	−0.06	−0.04
BE	0.15^#^	0.19*	−0.02	0.07
UN	−0.07	−0.16^#^	−0.08	−0.18*
Mean	0.01	0.00	0.01	0.00

^#^p < 0.10, *p < 0.05.

*SD, Self direction; ST, Stimulation; HE, Hedonism; AC, Achievement; PO, Power; SE, Security; TR, Tradition; CO, Conformity; BE, Benevolence; UN, Universalism*.

**Table 11:** Overall correlation matrix comparing values across data modalities in Study 3.

		**Raw text**										**Ipsatized text**									
		**SD**	**ST**	**HE**	**AC**	**PO**	**SE**	**TR**	**CO**	**BE**	**UN**	**SD**	**ST**	**HE**	**AC**	**PO**	**SE**	**TR**	**CO**	**BE**	**UN**
Raw rating	SD	0.02	0.03	0.10	0.06	−0.15	−0.09	−0.02	0.01	0.11	0.02	0.02	0.03	0.12	0.09	−0.16	−0.15	−0.02	0.01	0.18	0.02
	ST	0.06	0.02	0.11	−0.02	−0.12	0.01	−0.07	0.02	0.15	0.10	0.03	−0.01	0.09	−0.09	−0.17	−0.04	−0.11	−0.01	0.17	0.09
	HE	−0.02	−0.09	−0.06	−0.03	−0.17	0.00	−0.02	0.00	−0.04	0.04	0.00	−0.08	−0.05	−0.02	−0.16	0.03	−0.01	0.02	−0.02	0.08
	AC	0.00	0.08	0.04	0.07	−0.04	0.10	0.00	0.10	0.18	0.11	−0.10	0.01	−0.04	0.01	−0.11	0.05	−0.05	0.05	0.16	0.05
	PO	−0.10	0.03	0.01	−0.03	−0.01	−0.08	−0.08	−0.09	0.03	0.00	−0.07	0.09	0.06	0.01	0.03	−0.05	−0.05	−0.06	0.12	0.06
	SE	0.06	0.07	0.02	0.11	0.05	−0.03	−0.09	−0.10	0.05	0.01	0.10	0.09	0.03	0.17	0.05	−0.03	−0.10	−0.13	0.09	0.01
	TR	0.05	0.08	0.02	0.04	−0.04	−0.02	0.04	−0.05	0.10	0.07	0.03	0.07	−0.01	0.02	−0.07	−0.06	0.03	−0.10	0.13	0.06
	CO	−0.06	−0.13	−0.03	−0.12	−0.06	0.01	−0.10	−0.03	0.05	0.02	−0.02	−0.10	0.01	−0.11	−0.02	0.08	−0.07	0.01	0.16	0.09
	BE	−0.13	0.05	0.09	0.05	−0.10	0.09	−0.07	0.06	0.15	0.10	−0.23	0.02	0.08	0.03	−0.13	0.09	−0.10	0.04	0.19	0.10
	UN	0.01	0.01	−0.09	0.08	0.04	0.07	0.20	0.11	−0.01	−0.07	−0.05	−0.03	−0.16	0.06	0.00	0.04	0.19	0.10	−0.08	−0.16
Ipsatized rating	SD	0.05	0.01	0.07	0.04	−0.06	−0.07	−0.02	0.10	0.03	0.03	0.04	−0.01	0.07	0.03	−0.08	−0.13	−0.03	0.11	0.01	0.02
	ST	0.09	0.07	0.09	−0.06	−0.10	−0.03	−0.03	−0.07	0.04	0.08	0.11	0.07	0.10	−0.10	−0.12	−0.06	−0.04	−0.11	0.04	0.10
	HE	0.01	−0.05	−0.02	−0.02	0.05	0.08	0.13	0.06	−0.06	−0.01	−0.03	−0.09	−0.06	−0.08	0.02	0.08	0.11	0.04	−0.15	−0.06
	AC	0.04	0.03	0.00	0.05	0.08	0.12	0.08	0.18	0.06	0.09	−0.07	−0.06	−0.10	−0.04	0.00	0.05	0.03	0.13	−0.05	0.00
	PO	−0.11	0.01	−0.04	−0.08	0.01	−0.12	−0.11	−0.13	−0.04	−0.06	−0.03	0.10	0.05	−0.01	0.09	−0.06	−0.06	−0.08	0.07	0.03
	SE	0.07	0.04	−0.05	0.07	0.07	−0.05	−0.15	−0.19	−0.04	−0.05	0.16	0.11	−0.01	0.16	0.12	−0.01	−0.13	−0.19	0.01	0.00
	TR	0.10	0.03	0.01	0.06	0.01	0.00	0.10	−0.06	0.10	0.01	0.10	0.00	−0.02	0.04	−0.02	−0.05	0.09	−0.11	0.11	−0.03
	CO	−0.03	−0.13	0.01	−0.12	−0.03	0.05	−0.05	−0.06	0.04	−0.02	0.01	−0.12	0.05	−0.13	0.00	0.12	−0.03	−0.04	0.12	0.02
	BE	−0.15	−0.01	−0.02	−0.01	−0.08	−0.02	−0.07	−0.05	−0.02	−0.03	−0.13	0.05	0.04	0.07	−0.03	0.06	−0.03	−0.01	0.07	0.04
	UN	0.04	−0.04	−0.08	0.11	0.09	0.05	0.15	0.15	−0.02	−0.08	−0.01	−0.10	−0.15	0.10	0.05	0.01	0.13	0.15	−0.12	−0.18

*Correlations above r = 0.16 are significant at p < 0.05*.

*SD, Self direction; ST, Stimulation; HE, Hedonism; AC, Achievement; PO, Power; SE, Security; TR, Tradition; CO, Conformity; BE, Benevolence; UN, Universalism*.

*The meaning of the color indicates the shaded entries show value-type matched correlations*.

Focusing on the similarity of the correlation matrices, the Mantel test suggested low similarity: *r* = 0.11, *p* = 0.31. This is similar to the results observed in Study 2 (Table 12).

**Table 12:** Overall similarity of the correlation matrices (using Mantel correlations).

	**Raw text**	**Ipsatized text**	**Raw rating**
Raw text			
Ipsatized text	0.37*		
Raw rating	0.11	−0.13	
Ipsatized rating	0.19	−0.08	0.77**

**Figure 4:**
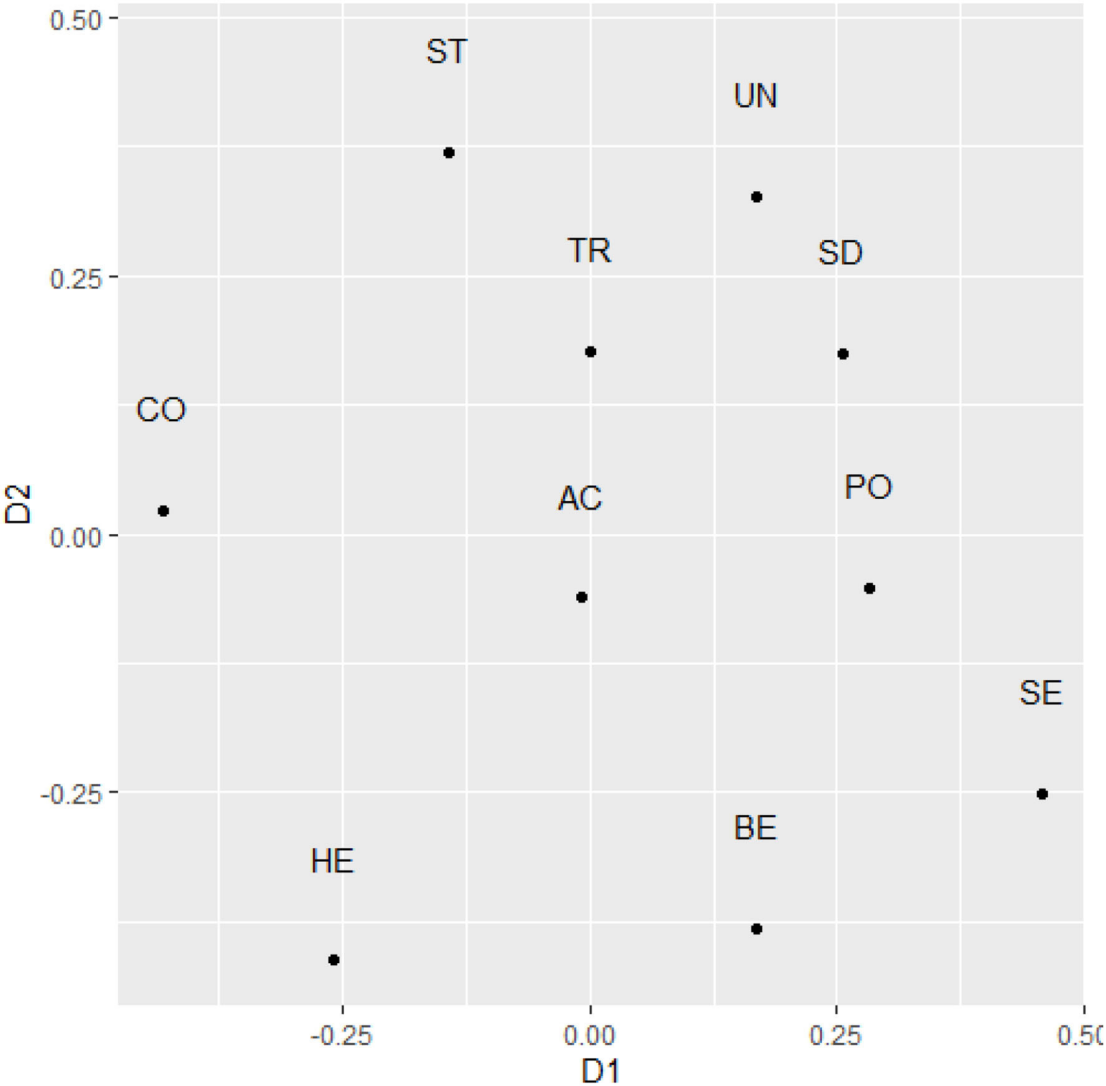
The two-dimensional representation of raw text-based value scores after rotation to maximal similarity with theoretical predictions in Study 3. SD, Self direction; ST, Stimulation; HE, Hedonism; AC, Achievement; PO, Power; SE, Security; TR, Tradition; CO, Conformity; BE, Benevolence; UN, Universalism.

Finally, examining the two-dimensional structure of the text-based scores, we found a stress-1 level of 0.18. Overall, the rotated dimensions showed relatively high level of congruence (0.861, Alienation coefficient: 0.509). However, an examination of the individual dimensions again suggested lower replicability of both dimension 1: *r* = 0.32 and dimension 2: *r* = 0.45. As in previous studies, Conservation values were relatively widespread across the two-dimensional representation. This time, Conformity and Security values were located at the opposing ends of dimension 1. Power and Achievement values were located relatively in the center of the two-dimensional structure. Benevolence values were located toward the same end as Achievement and Power values, which is not aligned with the theory (Figure 4).

## Overall Discussion

Our aim was to examine the extent to which value information can be extracted from narratives, both autobiographical and narratives based on ambiguous stimuli. For the autobiographical narratives, we found average correlations on matched value types between self-report survey responses and text-based value scores that are aligned with previously reported validation data (Ponizovskiy et al., 2020). In contrast, for the ambiguous stimuli narratives, we found few conceptually consistent correlations. When examining the overall pattern, only in Study 1 we observed a significant positive correlation of the correlation matrices (involving both diagonal and off-diagonal entries in the correlation matrices). When focusing on the content-matched value correlations (the diagonal elements only), overall, the autobiographical stories showed convergence at a similar level as the validation studies of Ponizovskiy et al. (the validities for texts in which participants had to describe their values were higher in the original studies). Therefore, extracting values from narratives, in particular from autobiographical narratives, appears feasible and may show some insights into salient values of individuals.

One interesting observation is that the most salient value types extracted from texts diverged somewhat from a universal hierarchy of values (Schwartz and Bardi, 2001) and also from our observed sample-specific value rating hierarchy. Achievement values emerged consistently within the top three most important values based on both autobiographical stories and stories based on ambiguous pictures. From a life history perspective, this may be understandable as autobiographical stories often revolve around resolving problems, typically within the parameters of the social norms set by the system and then told to be understandable within the local social and cultural context (McAdams and Pals, 2006). Hence, Achievement values may feature more saliently in these stories. It is noteworthy that these values were also quite salient in the ambiguous picture stories. One interpretation could be that Achievement values are salient for young adults attending university, which therefore makes them more salient when being presented with ambiguous social situations in those pictures. This clearly needs further exploration.

Focusing on the convergence in value means, Benevolence values were the most salient in both the text-based scores and the self-ratings. Benevolence values consistently emerge as the most important values in self-report ratings across different samples around the world, which highlights the importance of socially focused values in human societies. This salience highlights the evolutionary importance of coordinating one's actions with ingroups of humans irrespective of culture (Fischer, 2017).

When focusing on the correlations between self-ratings and textual ratings, computing the sample-size weighted average across the two autobiographical tasks, the strongest correlation was observed for Universalism values (average *r* = 0.19), followed by Stimulation (average *r* = 0.15), Power (average *r* = 0.13), Benevolence (average *r* = 0.12), Tradition (average *r* = 0.11) and Achievement (average *r* = 0.11). The least correlated values were Security values (average *r* = 0.01), Conformity (average *r* = 0.05), Self-Direction (average *r* = 0.07) and Hedonism values (average *r* = 0.08). In Ponizovskiy et al. (2020) validation study, the strongest correlations in the Facebook study were observed for Tradition and Universalism values, which were nevertheless somewhat weaker than the average correlations that we found. Hence, social values in both Facebook posts and autobiographical stories seem to converge relatively more strongly than person-focused values with self-ratings. At the same time, the pattern of the strength of correlations was not that clear. Future studies need to examine whether the focus of the stimulus material (e.g., blog posts vs. diaries) and the content of the material (e.g., type of blog content, specific autobiographical stories) may result in stronger or weaker convergence with self-report ratings.

Concerning the use of autobiographical stories vs. stories created based on ambiguous stimuli, we found weaker convergence between text-based value scores and self-ratings in the latter type of texts. This may not be surprising to researchers within the implicit motive tradition, who have long argued that ratings and implicit motives may not converge (Schultheiss and Brunstein, 2001; Hofer et al., 2006). Our contribution is that we used a theory-driven dictionary approach which does not require the more extensive coding of implicit motives or values (Suedfeld et al., 2011). If the task was to assess values from narratives that would be more in line with explicit self-ratings, our recommendation would be to elicit autobiographical or life-story type narratives instead of asking individuals to produce stories based on ambiguous stimuli. On the other hand, based on the implicit-motives research tradition, the potential unique contribution of scores derived from ambiguous stimuli over and above explicit self-report ratings may merit further research.

Extending this point, all these patterns of validity judged against self-ratings raise the well-known ground-truth problem when examining textual data (Boyd et al., 2021). A critical perspective on our findings could be that we report null results. Our view is that the results, to some large extent, reflect the state of the literature. We already mentioned the relative patterns observed in the original value dictionary study. When examining some of the classic personality studies within the personality literature, similarly weak results emerge. For example, associations between self-report personality scores and linguistic inquiry and word count (LIWC) (Chung and Pennebaker, 2008) dictionary scores for Facebook status updates tend to be in the range of −0.19 (association between Agreeableness and Anger) and 0.15 (association between Neuroticism and Negative emotion) (Schwartz et al., 2013). Most of the associations are close to zero. An analysis of personal blogs (Yarkoni, 2010), again correlating dictionary based scores from LIWC with self-ratings for personality showed an average absolute correlation of 0.078. Shifting to values, even topics extracted bottom up from the text did not converge consistently more strongly with self-ratings, with most associations falling below *r* = 0.10 threshold (Boyd et al., 2015). Returning to the ground-truth argument, it could be questioned whether text-based scores should be validated against self-report ratings: after all, text-based scores are behavior-based and do not suffer from many of the known problems of survey-based methods, such as acquiescence biases. There is relatively little research that has correlated value ratings with actual behavioral scores, that is observations or recordings of a real behavior which is not based on self-reports (Fischer and Karl, 2020). Online behavior has been suggested as providing an opportunity for capturing behavioral data, but any online behavioral correlates are not free of artifacts (Kosinski et al., 2015). In the absence of other validated methods, the most straightforward comparison remains the use of self-report scores.

Finally, focusing on both the value structures that emerge based on narratives (the multidimensional scaling results) and the overall patterns of correlations observed in Mantel's test, it becomes clear that the two-dimensional representations statistically diverge from the ideal structure that was based on self-report data (Schwartz, 1992). In other words, self-report values and text-based values appear to capture somewhat different motivational structures. Qualitatively, it is possible to discern motivational distinctions in these graphs (e.g., socially vs. personally focused values). However, the exact position diverges from the theoretical predictions. In particular, values along the self-transcendence vs. self-enhancement dimension emerged more closely to each other than what would be expected theoretically and values within the conservatism value cluster often emerged at opposing ends of the two-dimensional representation. As autobiographical narratives (and stories produced in response to ambiguous stimuli) are situation-specific, it is well plausible that these scores are more aligned with value states, which have received recent attention (Skimina et al., 2018, 2021). It is plausible that within specific situations, self-transcendence and self-enhancement values may not be compatible, with either one or the other value being salient within a situation. Due to the different nature of specific situations or events that are being recounted in these autobiographical stories, across these stories both values might be quite prominent (due to the different situations), which now leads to a different structure. Similarly, conservative values, such as those values emphasizing security and either following tradition or conforming to social norms may clash in specific situations. Our data did not allow us to focus on the structural properties within each narrative. This is clearly an avenue for further research.

Another avenue for future research is a closer examination of the interpretation of each term within the value dictionaries. Word choice, like behavior, is multidetermined and can be interpreted in different ways. Even when we only examine value ratings as predictors of word choice, the relations might be more complex than the circular model would imply. On the other hand, even within survey scales, the word choices may matter because value terms have complex interpretations, and this can shift interpretations. Interpretations of value terms may have played a significant role in previous observations of the divergence of individual vs. country-level value structures (Schwartz, 2006; Fischer et al., 2010; Fischer, 2012). Lexical analyses of value terms in dictionaries may provide novel avenues by opening options for word embeddings that can trace the conceptual space that is invoked by individual's value terms. Hence, the divergence in structure in autobiographical narratives observed in our current study fits a larger pattern of possible meaning shifts across levels of analysis (situation to individual to culture). Hence, a more focused linguistic analysis of value dictionaries relevant for individuals, subgroups, and cultures may provide novel insights for value researchers.

## Limitations

In addition to the points for future research directions already mentioned, one further limitation is that our samples were restricted to young adults attending introductory psychology classes in a Western society. Future studies should expand sampling to reach more diverse populations. A second limitation is that participants responded to short prompts in writing. Richer material might be obtained in the context of an in-depth interview. We used the writing tasks that have been used in previous studies (Rubin et al., 2009), but we encourage researchers to explore options to record verbal narratives and stories. Longer autobiographical narratives (e.g., book-length autobiographies) may also provide richer and more nuanced material for analyzing individuals at a distance. A third issue with any kind of textual analysis is that value terms in general or words from specific value domains may have different natural frequencies with which they are being used within a language. The fact that the dictionary is not balanced across values may further aggravate any hidden frequency issues. We adjusted both for the total number of value words and for total words (in Study 1). The convergence of these adjusted scores with ratings was lower. However, given the imbalanced nature of the dictionary and the unknown behavioral validity of both the value self-ratings and text-extracted value scores, we are in no position to resolve this issue. Future studies may need to carefully calibrate word-frequency effects across different types of populations and texts. A further limitation is that the narrative tasks were always presented prior to the self-report surveys. Our rationale for this order was not to prime individuals with value-specific content when writing their autobiographical stories or responding to ambiguous stimuli. Future studies should counterbalance the administration of these tasks to examine possible ordering effects. The choice of the value survey to be used may also warrant further attention. We used the most recently developed version of the Portrait Value Questionnaire (Schwartz et al., 2012). Other value instruments are available, and it may be useful to explore the ground truth problem with different surveys. Finally, as we noted above, text-based correlations with self-reports tend to be relatively weak and therefore, statistical significance requires larger sample sizes. This may be of particular relevance for clinical or organizational studies where participant pools may be restricted and therefore correlations between text-based scores and self-reports may not reach statistical significance due to power limitations.

## Conclusions

We extended previous research by demonstrating that it is possible to apply value dictionaries to autobiographical narratives to extract personal value information. In line with previous research, we found small but consistent correlations between values that are matched by motivational content. This is promising in that it highlights that it is possible to capture values from even short autobiographical stories, which makes value assessment at a distance (e.g., interviews and biographies) feasible. The overall structures nevertheless diverged somewhat both from self-ratings and from theoretical expectations, highlighting that further research is needed that examines value content in the context of life stories.

## Data Availability Statement

The datasets presented in this study can be found in online repositories. The names of the repository/repositories and accession number(s) can be found below: https://osf.io/gqmr4/.

## Ethics Statement

The studies involving human participants were reviewed and approved by the School of Psychology Human Ethics Committee under delegated authority of Victoria University of Wellington's Human Ethics Committee (ID0000023640), protocol number: RMP0000028177. The patients/participants provided their written informed consent to participate in this study.

## Author Contributions

RF developed the study idea and wrote the first draft. JK and RF collected the data. JK, ML-R, and RF analyzed the data. RF, VF, and JK interpreted the results. All authors contributed to the study design, provided critical feedback, and comments. All authors contributed to the article and approved the submitted version.

## Conflict of Interest

The authors declare that the research was conducted in the absence of any commercial or financial relationships that could be construed as a potential conflict of interest.

## Publisher's Note

All claims expressed in this article are solely those of the authors and do not necessarily represent those of their affiliated organizations, or those of the publisher, the editors and the reviewers. Any product that may be evaluated in this article, or claim that may be made by its manufacturer, is not guaranteed or endorsed by the publisher.
