# Supplementary material for: Opportunities and Challenges of Extracting Values in Autobiographical Narratives
Source: Front Psychol. 2022 Aug 2;13:886455. doi: 10.3389/fpsyg.2022.886455 (PMC9379099; doi:10.3389/fpsyg.2022.886455)

Supplement

**The opportunities and challenges of extracting values in autobiographical narratives**

Ronald Fischer^1,2^, Johannes Karl^1^, Velichko Fetvadjiev^3,4^, Adam Grener^1^, Markus Luczak-Roesch^1^

^1^ Victoria University of Wellington, New Zealand
^2^ Instituto D’Or de Pesquisa e Ensino, Rio de Janeiro, Brazil
^3^ University of Amsterdam, The Netherlands

^4^ North-West University, South Africa

SFigure 1. Bi-term topic model for Positive event


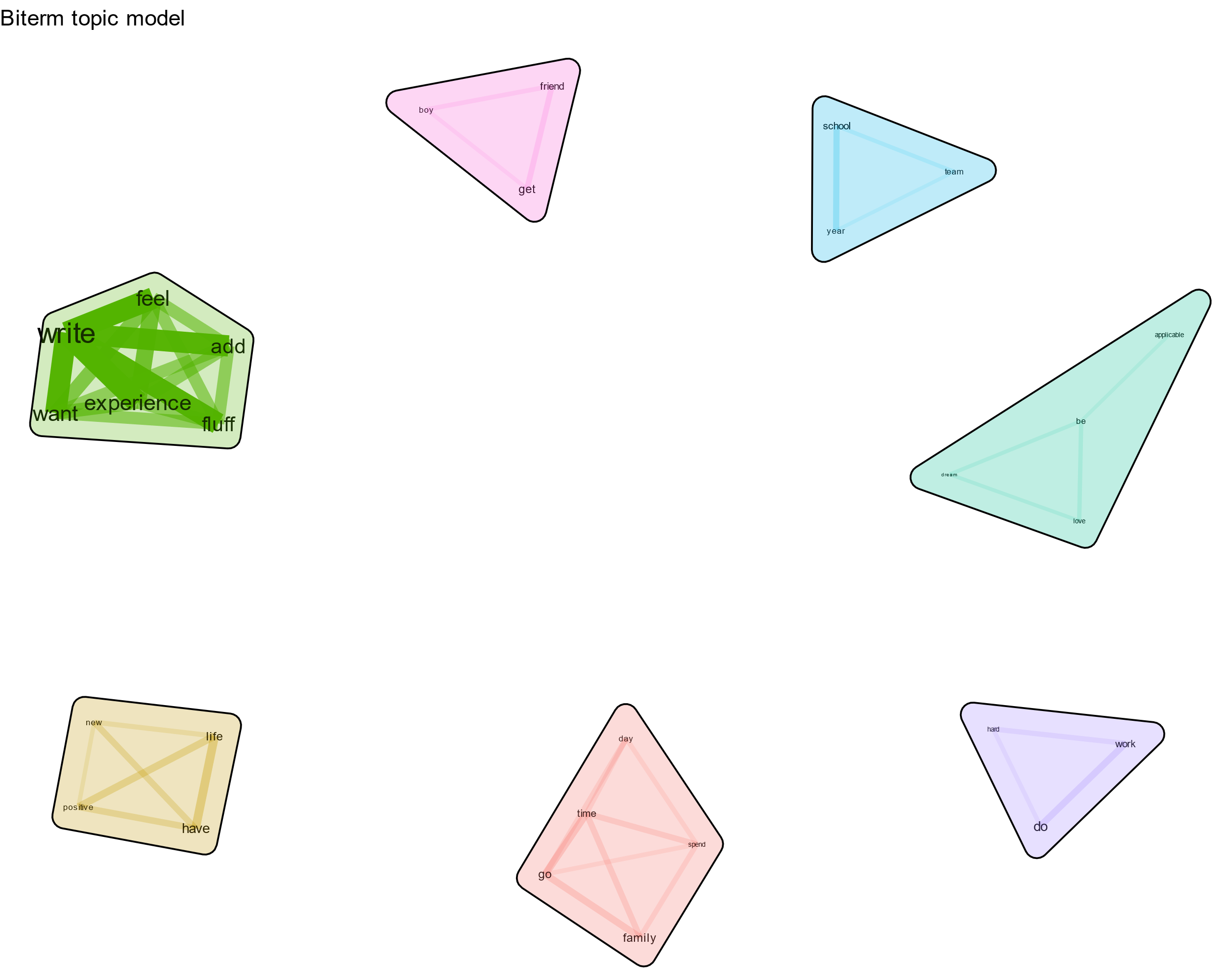


SFigure 2. Bi-term topic model for challenging event


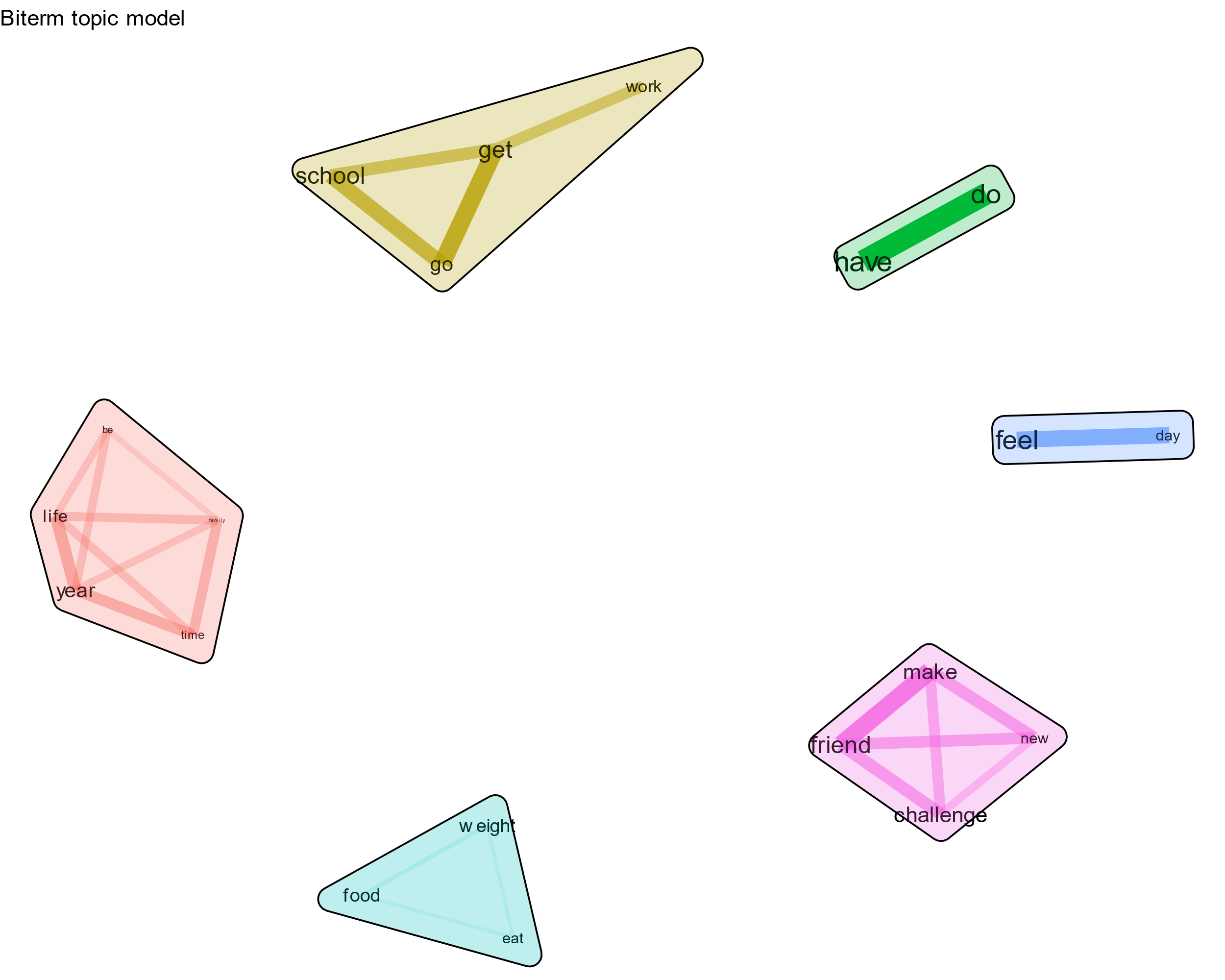


SFigure 3. Bi-term topic model for first event mentioned (Study 2)


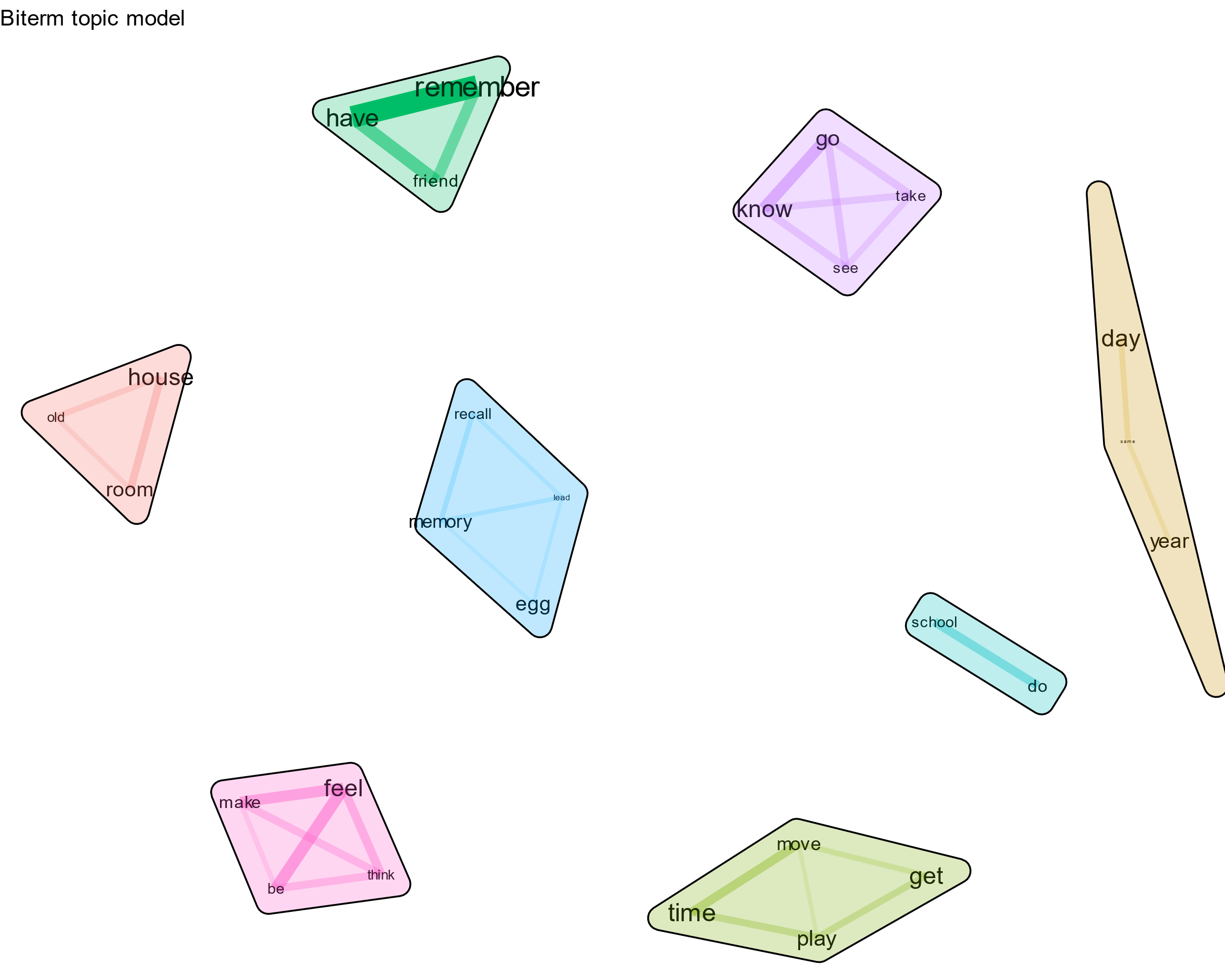


SFigure 4: Bi-term topic model for second event mentioned


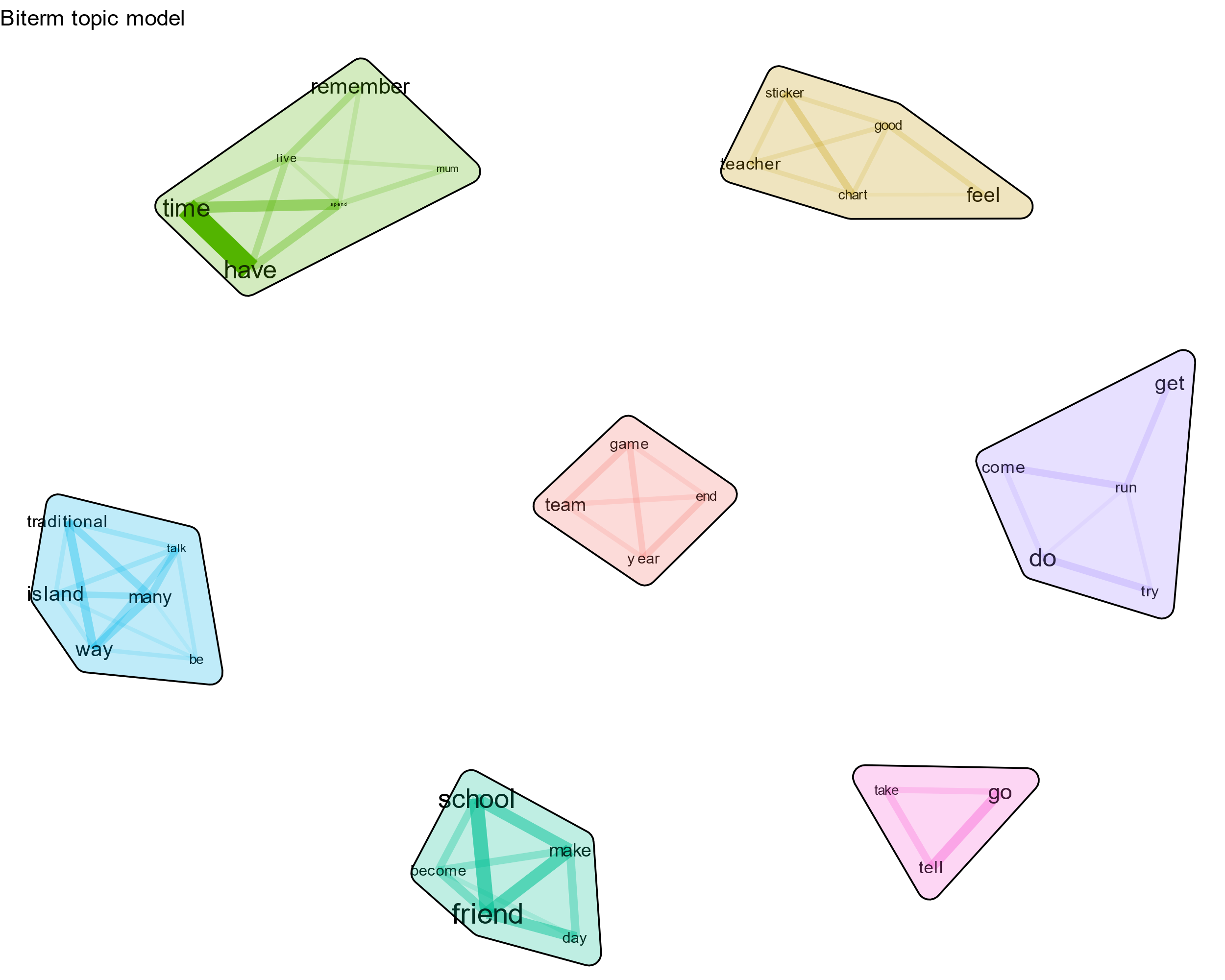


SFigure 5: Bi-term topic model for third event mentioned


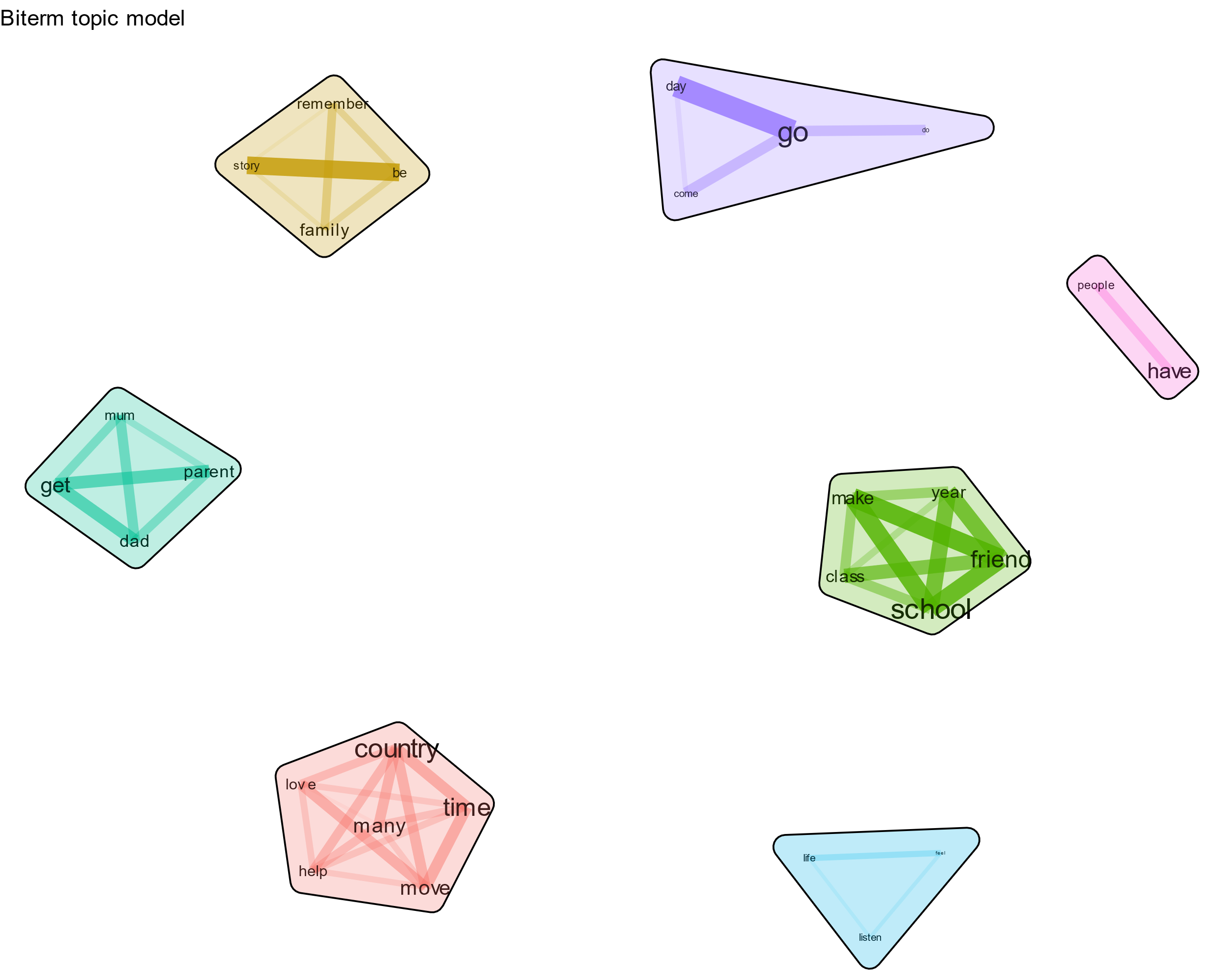


SFigure 6: Bi-term topic model for forth event mentioned


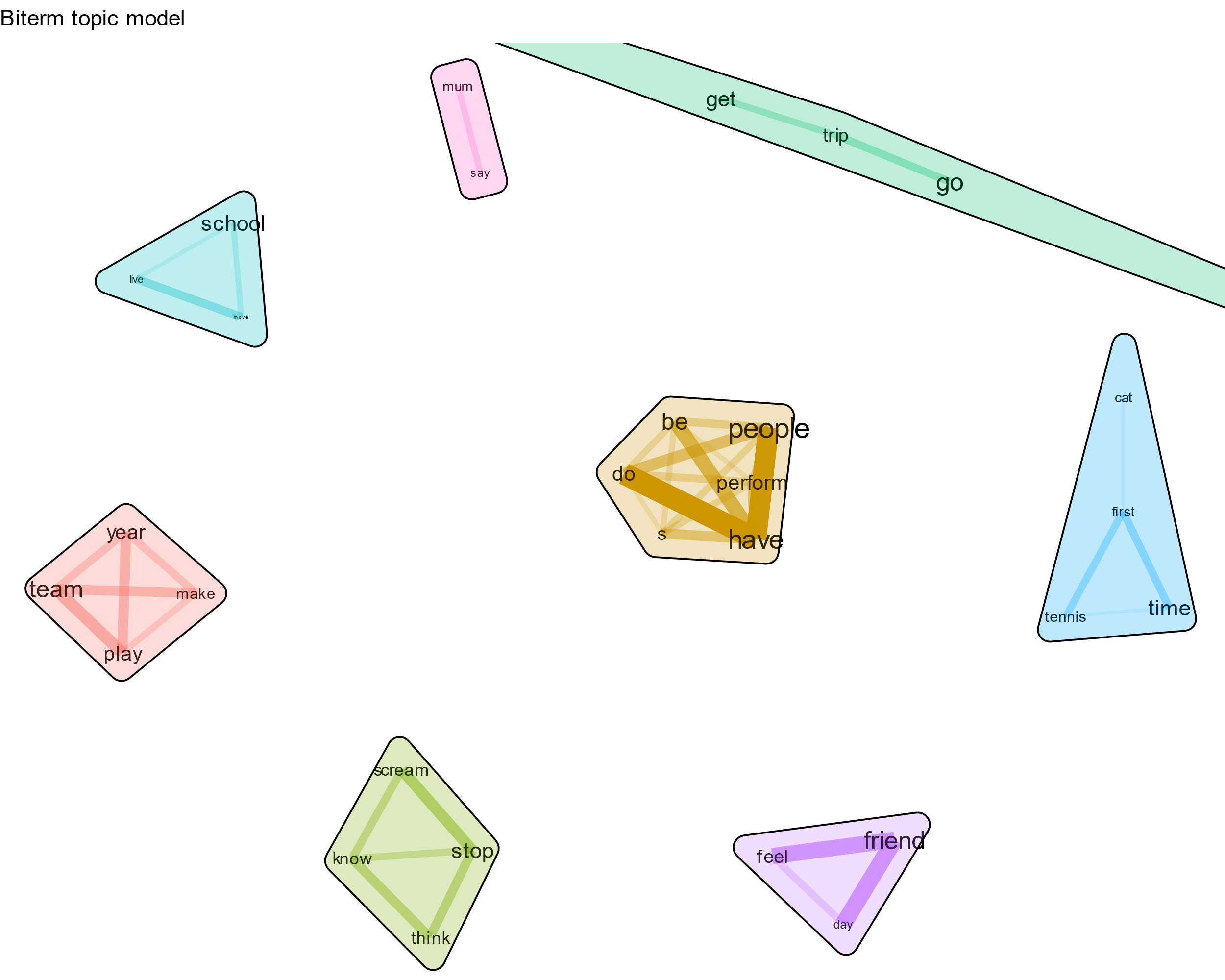


SFigure 7: Bi-term topic model for fifth event mentioned


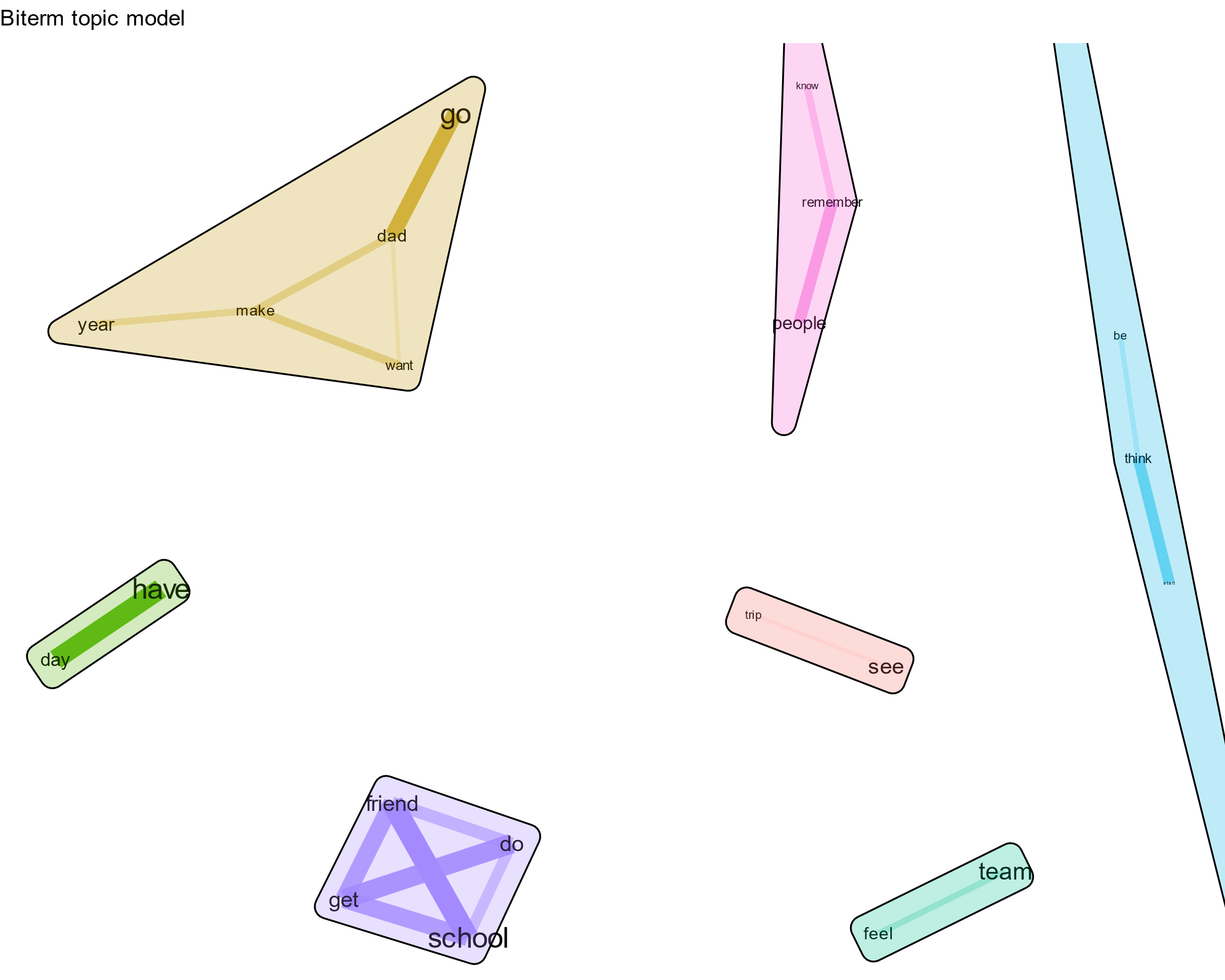


SFigure 8: Bi-term topic model for the sixth event mentioned


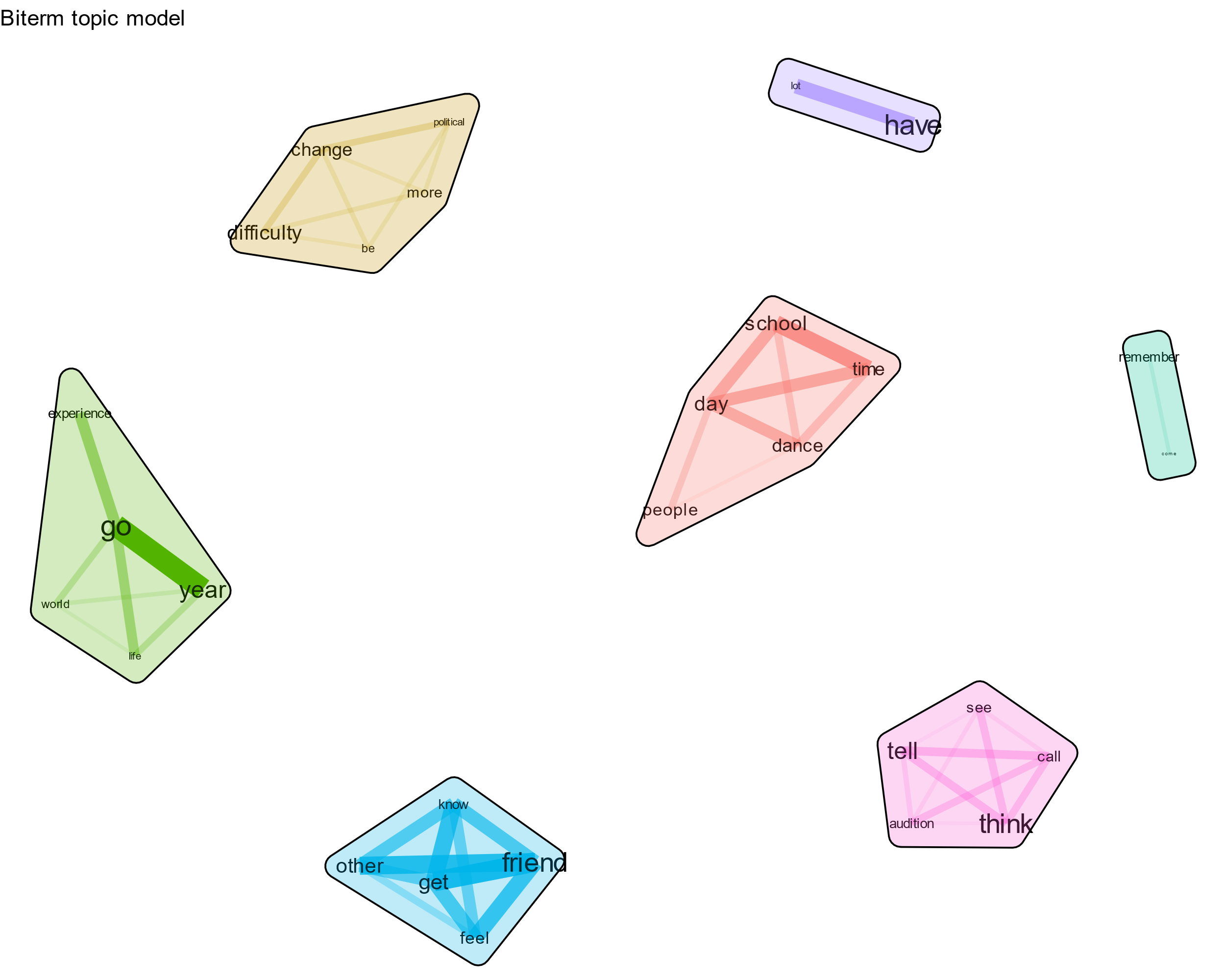


SFigure 9: Bi-term topic model for the seventh event mentioned


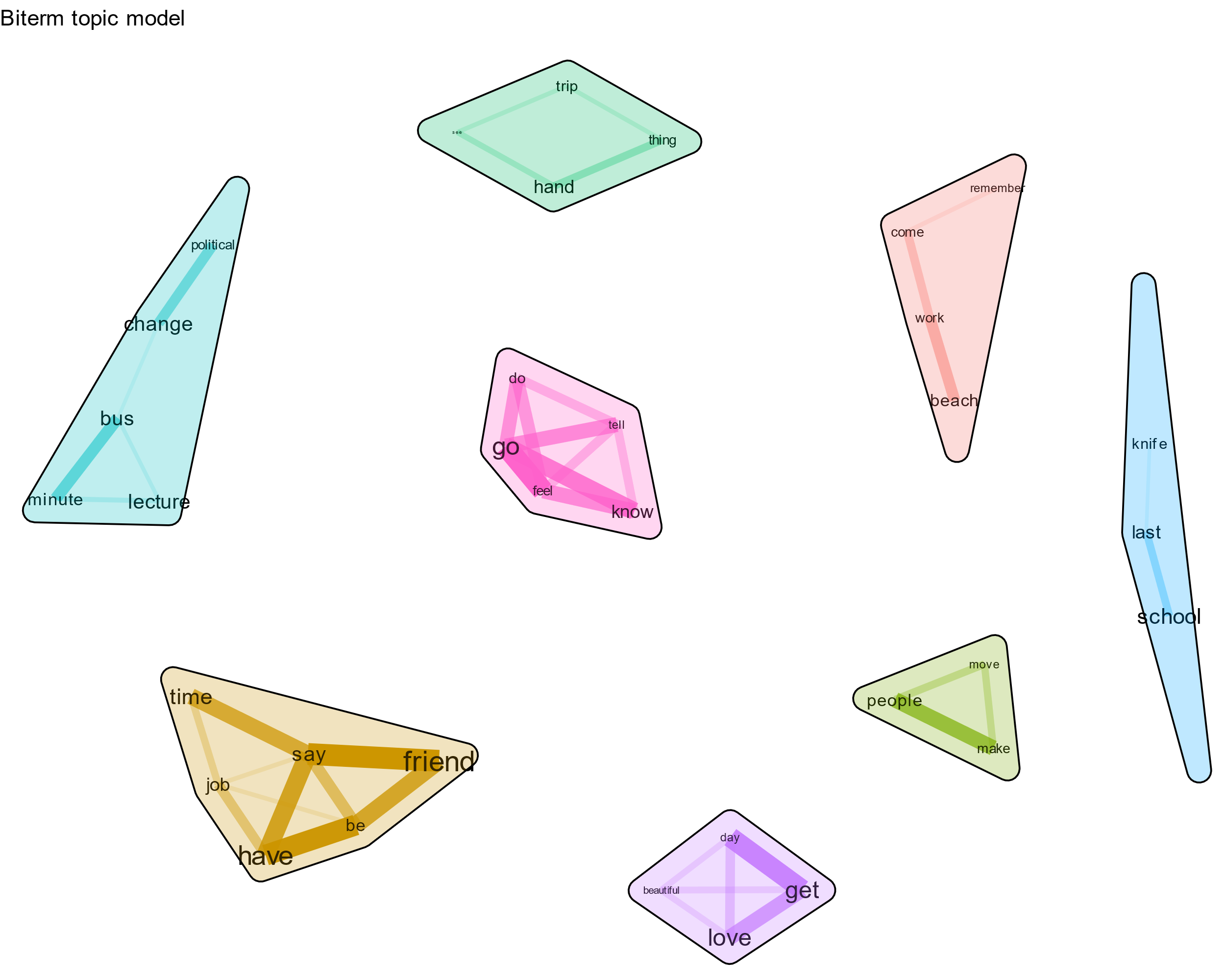

Supplement: Supplementary file 1 [file Table_1.DOCX]
